# Caregiver-Focused, Web-Based Interventions: Systematic Review and Meta-Analysis (Part 2)

**DOI:** 10.2196/11247

**Published:** 2018-10-26

**Authors:** Jenny Ploeg, Muhammad Usman Ali, Maureen Markle-Reid, Ruta Valaitis, Amy Bartholomew, Donna Fitzpatrick-Lewis, Carrie McAiney, Diana Sherifali

**Affiliations:** 1 Aging, Community and Health Research Unit School of Nursing, Faculty of Health Sciences McMaster University Hamilton, ON Canada; 2 Department of Health, Aging and Society McMaster University Hamilton, ON Canada; 3 McMaster Institute for Research on Aging McMaster University Hamilton, ON Canada; 4 Faculty of Health Sciences School of Nursing McMaster University Hamilton, ON Canada; 5 Department of Health Research Methods, Evidence and Impact Faculty of Health Sciences McMaster University Hamilton, ON Canada; 6 World Health Organization Collaborating Centre for Primary Care and Health Human Resources Hamilton, ON Canada; 7 Department of Family Medicine McMaster University Hamilton, ON Canada; 8 Program for Interpersonal Practice, Education and Research Department of Psychiatry and Behavioural Neurosciences McMaster University Hamilton, ON Canada; 9 McMaster Evidence Review and Synthesis Team School of Nursing, Faculty of Health Sciences McMaster University Hamilton, ON Canada; 10 Diabetes Care and Research Program Hamilton Health Sciences Hamilton, ON Canada

**Keywords:** burden, caregivers, chronic conditions, education, internet, meta-analysis, support, Web-based interventions

## Abstract

**Background:**

Approaches to support the health and well-being of family caregivers of adults with chronic conditions are increasingly important given the key roles caregivers play in helping family members to live in the community. Web-based interventions to support caregivers have the potential to lessen the negative health impacts associated with caregiving and result in improved health outcomes.

**Objective:**

The primary objective of this systematic review and meta-analysis was to examine the effect of caregiver-focused, Web-based interventions, compared with no or minimal Web-based interventions, on caregiver outcomes. The secondary objective was to assess the effect of different types of Web-based interventions (eg, education, peer and professional psychosocial support, and electronic monitoring of the care recipient), compared with no or minimal Web-based interventions, on caregiver outcomes.

**Methods:**

MEDLINE, EMBASE, CIHAHL, PsychInfo, Cochrane, and AgeLine were searched from January 1995 to April 2017 for relevant randomized controlled trials (RCTs) or controlled clinical trials (CCTs) that compared caregiver-focused, Web-based intervention programs with no or minimal Web-based interventions for caregivers of adults with at least one chronic condition. Studies were included if they involved: adult family or friend caregivers (aged ≥18 years) of adults living in the community with a chronic condition; a caregiver-focused, Web-based intervention of education or psychosocial support or electronic monitoring of the care recipient; and general caregiver outcomes (ie, burden, life satisfaction, self-efficacy or mastery, reaction to problem behavior, self-esteem, strain, and social support). Title and abstract as well as full-text screening were completed in duplicate. Data were extracted by a single reviewer and verified by a second reviewer, and risk of bias assessments were completed accordingly. Where possible, data for these caregiver outcomes were meta-analyzed.

**Results:**

The search yielded 7927 unique citations, of which 294 studies were screened at full text. Of those, 14 studies met the inclusion criteria; 12 were RCTs and 1 study was a CCT. One study used an RCT design in 1 country and a CCT design in 2 other countries. The beneficial effects of any Web-based intervention program, compared with no or minimal Web-based intervention, resulted in a mean increase of 0.85 points (95% CI 0.12 to 1.57) for caregiver self-esteem, a mean increase of 0.36 points (95% CI 0.11 to 0.62) for caregiver self-efficacy or mastery, and a mean decrease of 0.32 points (95% CI −0.54 to −0.09) for caregiver strain. However, the results are based on poor-quality studies.

**Conclusions:**

The review found evidence for the positive effects of Web-based intervention programs on self-efficacy, self-esteem, and strain of caregivers of adults living with a chronic condition. Further high-quality research is needed to inform the effectiveness of specific types of Web-based interventions on caregiver outcomes.

**Trial Registration:**

PROSPERO CRD42018091715; https://www.crd.york.ac.uk/prospero/display_record.php?RecordID=91715 (Archived by WebCite at http://www.webcitation.org/738zAa5F5)

## Introduction

The number of individuals living with chronic conditions is on the rise globally [1]. Family and friend caregivers provide up to 75% of the health and supportive care needs for older adults living in the community in Canada [2]. Although caregiving can be very rewarding, it is also associated with adverse physical, mental, and psychosocial health outcomes [3-5]. Examples of negative outcomes as a result of caregiving include burden, strain, being dissatisfied with life, feeling alone or isolated, and having low self-efficacy [3-8]. Thus, practical solutions to address the needs of caregivers are urgently needed.

Recently, there has been great interest in the use of Web-based interventions to support caregivers. It has been suggested that the delivery of health care interventions through the Web may result in improved accessibility of services as well as reduced health care costs [9]. There is accumulating evidence for the positive effect of caregiver-focused, Web-based interventions in 11 recent systematic or narrative reviews [10-20]. All of these reviews provided some evidence of improvements in caregivers’ health or well-being (eg, burden, depression, self-efficacy, and confidence) as a result of Web-based programs. Most reviews included studies with both high- and low-quality designs and noted the limited methodological quality of included studies as a concern. In addition, most reviews did not examine the effect of different types of Web-based support on caregivers. Finally, none of the reviews included a meta-analysis to quantify the magnitude of effect across studies.

The primary objective of this study was to conduct a systematic review and meta-analysis to assess the effect of caregiver-focused, Web-based interventions, compared with no or minimal web-based interventions, on outcomes for caregivers of adults with at least one chronic condition living in the community. The caregiver outcomes examined in this paper include burden, life satisfaction, self-efficacy or mastery, reaction to problem behavior, self-esteem, strain, and social support. The secondary objective was to examine whether specific types of Web-based interventions had a beneficial effect on these caregiver outcomes, to address previous review limitations. Of note, this review included only studies with the most rigorous designs, randomized controlled trials (RCTs) and controlled clinical trials (CCTs). This is a companion paper to a systematic review and meta-analysis that examines the effect of internet-based interventions on caregiver mental health outcomes [21].

## Methods

### Reporting Guidelines

This systematic review and meta-analysis was conducted following the Preferred Reporting Items for Systematic Reviews and Meta-Analysis guidelines [22].

### Population

The population of interest included family and friend caregivers, aged ≥18 years, who were providing caregiving support to adults (≥18 years) living in the community with at least one chronic condition (ie, “care recipient”).

### Interventions

Studies selected for this systematic review included those that examined any caregiver-focused, Web-based modality to deliver an intervention, which could include either a single component program or multimodal program.

### Outcomes

The outcomes assessed in this meta-analysis included the following caregiver outcomes: burden, life satisfaction, self-efficacy or mastery, reaction to problem behavior, self-esteem, strain, and social support. Mental health outcomes are addressed in a companion paper [21].

### Study Design

#### Inclusion and Exclusion

Studies were included if they met the following inclusion criteria: study designs were an RCT or CCT; studies examined any Web-based intervention program for caregivers of older adults having at least one chronic condition and living in the community; studies were published between January 1, 1995 and April 19, 2017; studies were published in English; studies reported on at least one caregiving outcome of interest (burden, life satisfaction, self-efficacy or mastery, reaction to problem behavior, self-esteem, strain, or social support); studies used any measurement tool to examine the outcomes of interest; and studies in which the control group received no or minimal Web-based intervention. Of note, there were no restrictions on the nature of chronic conditions of care recipients. The exclusion criteria included all other types of study designs (ie, observational studies and case reports), gray or unpublished literature, conference abstracts, and letters or editorials. Furthermore, all published study protocols without preliminary results for data extraction were also excluded.

#### Search Strategy

A peer-reviewed search strategy was developed by 2 research librarians at McMaster University. EMBASE, MEDLINE, PsychInfo, CINAHL, Cochrane, and AgeLine were searched for studies published between January 1, 1995 and April 19, 2017. In addition, reference lists of systematic reviews were searched for relevant studies not captured by the initial search. Results were deduplicated, and the citations were uploaded to a secure Web-based platform. Multimedia Appendix 1 provides detailed information about the search terms.

#### Selection of Studies

Two reviewers independently selected studies for possible inclusion based on a title and abstract review. Studies meeting the inclusion criteria by either reviewer then underwent full-text review. Any disagreements were discussed between reviewers, and a third party was involved to help reach consensus, as necessary.

### Data Extraction and Quality Assessment

Full-data extraction, including characteristics of included studies, was completed by one reviewer and verified by a second reviewer. The risk of bias (RoB) found in individual studies was assessed by one reviewer and verified by a second reviewer. RoB was assessed using the Cochrane RoB framework [23], which evaluates the level of bias for sequence generation, allocation concealment, blinding, completeness of outcome assessment, selective reporting, and other biases. The quality of the clinical evidence was critically appraised by one reviewer and verified by a second reviewer using the Grading of Recommendations Assessment, Development, and Evaluation system (GRADE), which evaluates the risk for bias, inconsistency, indirectness, and imprecision for each outcome [24]. Disagreements were resolved through consensus between the 2 reviewers.

### Data Analysis

We used a meta-analysis to combine the results across studies for each outcome using the published data from included studies. To perform the meta-analysis, we used immediate posttreatment data (mean, SD) for continuous outcomes such as burden, life satisfaction, self-efficacy or mastery, reaction to problem behavior, self-esteem, strain, and social support. In addition, we used intention-to-treat outcome data where possible; however, if no intention-to-treat data were reported, we used outcome data obtained from those who completed the study.

The DerSimonian and Laird random-effects models with the inverse variance method were used to generate the summary measures of effect in the form of standardized mean difference (SMD) [25]. SMD accounts for similar outcomes measured using different assessment tools (eg, caregiver burden assessed using different outcome measures such as the Zarit Burden Interview and Caregiver Quality of Life Scale). In this situation, it was necessary to standardize the results of the studies to a uniform scale before they could be combined in quantitative synthesis. SMDs were calculated using change from the baseline data for intervention and control groups for each study with relevant outcome data. For each outcome, data from the corresponding study were used to calculate the mean difference between pretreatment (baseline) and posttreatment (final or endpoint) values along with its SD for both intervention and control groups. In studies where SD was not reported, we calculated it from the reported SE of the mean, 95% CIs and *P* values, or *z* values using equations provided in Chapters 7 and 9 of the Cochrane Handbook for Systematic Reviews of Interventions [26,27]. SMD is interpreted on the basis of its magnitude according to Cohen *d* recommended thresholds (~0.2, small effect; ~0.5, medium effect; and ~0.8, large effect) [28].

The primary meta-analysis examined caregiver-focused, Web-based interventions by caregiver outcome. Subsequently, the secondary meta-analysis examined the effects of specific types of caregiver-focused, Web-based intervention programs on caregiver outcomes. Based on our previous work [18], intervention types were categorized accordingly: Web-based information or education only; Web-based information or education plus peer psychosocial support; Web-based information or education plus professional psychosocial support; Web-based information or education plus combined peer and professional psychosocial support; and Web-based information or education plus professional psychosocial support plus electronic monitoring of the care recipient.

The statistical heterogeneity of combined studies was examined using standard methods. The *I*^2^ statistic was used to quantify the magnitude of statistical heterogeneity between studies where *I*^2^ of 30%-60% represents moderate and *I*^2^ of >60% represents substantial heterogeneity [26]. We used *P*<.10 as a guide to indicate where statistically significant heterogeneity may exist, upon which a closer examination of study differences was performed. All analyses were performed using Review Manager (RevMan Version 5.3, The Nordic Cochrane Centre, The Cochrane Collaboration, Copenhagen, Denmark) [29], STATA (version 14; Stata Corp, College Station, Texas, USA) [30], and GRADEpro Guideline Development Tool software packages [31].

## Results

### Search

**Figure 1:**
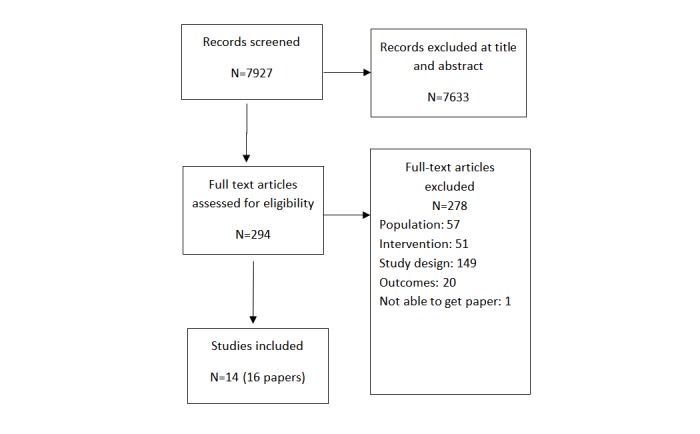
Flow diagram for the study selection of Web-based interventions on caregiving outcomes.

The search resulted in 7927 unique citations, which were screened independently by 2 project staff, as seen in Figure 1. At title and abstract screening, we excluded 7633 studies, leaving 294 studies to be screened at full text. Of these, we identified 14 studies (16 papers) that met the inclusion criteria for this review. References lists of the on-topic systematic reviews and included studies were searched, but no additional studies were added.

### Summary of Included Studies

Multimedia Appendix 2 presents the purpose, methods, participants, and intervention of the included studies.

### Study Design

#### Type of Studies

Among 14 included studies, 12 were RCTs [32-43], 1 was a CCT [44], and 1 study used an RCT design in 1 country and a CCT design in 2 other countries [45]. Companion papers [46,47]were included for the studies by DuBenske [37] and Smith [41] respectively. Of 12 RCTs, 4 were conducted in Europe [33-36], 7 in the United States [32,37,38,40-43], and 1 in South Korea [39]. The one CCT was conducted across the United States, Puerto Rico, and Mexico [44], and the study that used both CCT and RCT designs was conducted across 3 European countries [45]. All included studies had relatively small sample sizes (≤150 subjects per arm) and most had a length of follow-up of ≤6 months. One study included a slightly longer study follow-up period of 1 year [42]. In addition, 7 of 14 studies included reference to a theoretical or conceptual framework for the intervention, including stress and coping [34,35,37,41,43], framework of systemic organization [42], and the concept of ambient assisted living [33].

#### Study Population

Most studies included caregivers aged ≥50 years (mean age ranged from 53.8-66.0) [32,34-39,41,42,45], except 1 study that included caregivers who were working and reported a slightly lower mean age of 46.9 years [43]. In addition, 2 studies did not provide information on the average age of caregivers [33,44], and 1 study reported that 40% were >50 years [40]. Next, 11 of 14 studies reported caregiver gender; in 10 studies, more than half of the caregivers were females (56.3%-100%). In relation to the type of chronic conditions among care recipients, 9 studies included persons with some form of dementia [32-36,38,43-45]. In 3 studies, care recipients were stroke survivors [39,41,42]. Care recipients in 1 study had nonsmall cell lung cancer [37] and in another study, care recipients had brain injury [40].

#### Type of Web-Based Intervention

Among 14 included studies, 3 studies used a *Web-based information or education only* intervention [38,40,43], 3 studies used a *Web-based information or education plus peer psychosocial support* intervention [33-35], 1 study used a *Web-based information or education plus professional psychosocial support* intervention [39], 6 studies used a *Web-based information or education plus combined peer and professional psychosocial support* intervention [32,36,37,41,42,44], and 1 study used a *Web-based information or education plus professional psychosocial support intervention plus electronic monitoring* [45].

#### Comparator Groups

The comparator groups received usual care or were part of a wait-list control wherein they had access to the Web-based program at the end of the study [32-36,39,42,43,45], had access to printed materials [44], or had access to a website with general information related to the condition or resources [37,38,40,41].

### Outcomes

Among 14 included studies, outcomes examined included the following: burden (n=5); life satisfaction (n=3); self-efficacy or mastery (n=9); reaction to problem behavior (n=2); self-esteem (n=1); strain (n=1); and social support (n=2). Measurement tools to assess caregiver outcomes varied across included studies (Multimedia Appendix 3).

Table 1 shows the results of the critical appraisal of individual studies for the level of bias for sequence generation, allocation concealment, blinding, completeness of outcome assessment, selective reporting, and other biases. Overall, the Cochrane RoB showed the mixed quality of study methodology; 1 study had low RoB [35], 4 studies had high RoB [34,37,44,45], and 9 studies had unclear RoB because of the lack of relevant details in the published papers [32,33,36,38-43].

### Effectiveness of Web-Based Interventions

The meta-analysis included an examination of any Web-based intervention as well as an examination of each type of Web-based intervention by caregiver outcome. Multimedia Appendix 4 shows all forest plots.

#### Any Web-Based Intervention

Table 2 summarizes the results of the meta-analysis of any caregiver-focused, Web-based intervention on caregiver outcomes. Compared with no or minimal Web-based intervention, any type of Web-based intervention resulted in a statistically significant mean increase of 0.85 points (95% CI 0.12 to 1.57) for caregiver self-esteem, 0.36 points (95% CI 0.11 to 0.62) for caregiver self-efficacy or mastery, and a decrease of 0.32 points (95% CI −0.54 to −0.09) for caregiver strain. There were no statistically significant differences between groups for the caregiver outcomes of caregiver burden, life satisfaction, reaction to problem behavior, and social support. In addition, heterogeneity for the combined effect estimate was observed for the outcomes of caregiver burden, self-efficacy or mastery, reaction to problem behavior, and social support. The overall GRADE quality of evidence for each outcome ranged from moderate to very low. See Multimedia Appendix 5 for the full GRADE assessment details.

#### Effect of Different Types of Web-Based Interventions

Caregiver outcomes of interest were examined for each type of Web-based intervention, as shown in Table 3. For *information or education only* interventions, results showed a significant reduction with small effect sizes in caregiver strain (1 study; SMD=−0.32, 95% CI −0.54 to −0.09, *P*=.007) and self-efficacy or mastery (1 study; SMD=0.31, 95% CI 0.08 to 0.53, *P*=.009). These results were based on the moderate quality of evidence. The remaining outcomes of life satisfaction and reaction to problem behavior, which were assessed in only one study each, did not show statistically significant differences between groups.

For studies that examined *information or education plus peer psychosocial support*, there were no differences between intervention and control groups for any of the outcomes, including burden, life satisfaction, self-efficacy or mastery, and reaction to problem behavior; the quality of this evidence was very low. For studies that examined *information or education plus professional psychosocial support*, results showed a mean increase of 1.2 points (95% CI 0.48 to 1.92) for self-efficacy or mastery compared with no or minimal Web-based intervention; the quality of this evidence was very low.

**Table 1:** Risk of bias (RoB) of included studies.

Author, year	Sequence Generation	Allocation Concealment	Blinding of Participants/ Providers	Blinding of Outcome Assessment	Incomplete Outcome Data	Selective Reporting	Other Bias	Overall RoB
Beauchamp et al, 2005 [43]	Unclear	Unclear	Unclear	Unclear	Low	Low	Low	Unclear
Cristancho-Lacroix et al, 2015 [34]	Low	Unclear	High	High	Low	Low	High	High
DuBenske et al, 2014 [37] (Companion Paper: Gustafson et al, 2013 [46])	Unclear	Unclear	High	High	High	Low	High	High
Hattink et al, 2015 [35]	Low	Unclear	Low	Low	Low	Low	Low	Low
Hattink et al, [45]	High	High	Unclear	Unclear	Low	Low	Low	High
Kajiyama et al, 2013 [38]	Unclear	Unclear	Unclear	Unclear	High	Low	Low	Unclear
Kim et al, 2013 [39]	Low	Unclear	High	Unclear	Low	Low	Low	Unclear
McLaughlin et al, 2013 [40]	Unclear	Unclear	Unclear	Unclear	Low	Low	Low	Unclear
Fowler et al, 2016 [32]	Low	Unclear	High	Unclear	Low	Low	Low	Unclear
Núñez-Naveira et al, 2016 [33]	Low	Unclear	Unclear	Unclear	Low	Low	Low	Unclear
Pagán-Ortiz et al, 2014 [44]	High	High	Unclear	Unclear	Low	Low	Unclear	High
Pierce et al, 2009 [42]	Unclear	Unclear	Unclear	Unclear	High	Low	Low	Unclear
Smith et al, 2012 [41] (Companion Paper: Steiner et al, 2002 [47])	Low	Unclear	Unclear	Low	Low	Low	Low	Unclear
Torkamani et al, 2014 [36]	Unclear	Unclear	Unclear	Unclear	Low	Low	Low	Unclear

**Table 2:** A summary of the effectiveness of any Web-based intervention.

Caregiver outcomes	Number of studies	Intervention, n	Control, n	Estimate standardized mean difference (95% CI)	I^2^ (%)	Grading^a^
Caregiver burden	5	132	147	0.03 (−0.31 to 0.36)	48	Very low
Life satisfaction	3	170	165	−0.17 (−0.39 to 0.04)	0	Very low
Self-efficacy or mastery	9	306	309	0.36 (0.11 to 0.62)	46	Low
Reaction to problem behavior	2	71	81	−0.10 (−0.66 to 0.45)	63	Very low
Self-esteem	1	15	17	0.85 (0.12 to 1.57)	N/A^b^	Very low
Caregiver strain	1	150	149	−0.32(−0.54 to −0.09)	N/A	Moderate
Social support	2	30	34	−0.38 (−1.12 to 0.35)	53	Very low

^a^Grading of recommendations assessment, development, and evaluation system quality assessment.

^b^N/A: not applicable.

**Table 3:** A summary of the effectiveness of types of Web-based interventions.

Caregiver outcomes		Number of studies	Intervention, n	Control, n	Estimate standard mean difference (95% CI)	I^2^ (%)	Grading^a^
**Information or education**						
	Life satisfaction	1	104	97	−0.22 (−0.50 to 0.06)	N/A^b^	Very low
	Self-efficacy or mastery	1	150	149	0.31 (0.08 to 0.53)	N/A	Moderate
	Reaction to problem behavior	1	46	57	−0.35 (−0.75 to 0.04)	N/A	Very low
	Strain	1	150	149	−0.32 (−0.54 to −0.09)	N/A	Moderate
**Information or education plus peer psychosocial support**						
	Burden	2	46	49	0.17 (−0.24 to 0.57)	0	Very low
	Life satisfaction	1	30	31	0.08 (−0.43 to 0.58)	N/A	Very low
	Self-efficacy or mastery	3	76	80	0.14 (−0.41 to 0.69)	66	Very low
	Reaction to problem behavior	1	25	24	0.22 (−0.34 to 0.78)	N/A	Very low
**Information or education plus professional psychosocial support**						
	Self-efficacy or mastery	1	18	18	1.20 (0.48-1.92)	N/A	Very low
**Information or education plus peer and professional psychosocial support**						
	Burden	3	86	98	−0.03 (−0.57 to 0.50)	67	Very low
	Life satisfaction	1	36	37	−0.24 (−0.70 to 0.22)	N/A	Very low
	Self-efficacy or mastery	3	45	47	0.52 (0.10-0.94)	0	Very low
	Self-esteem	1	15	17	0.85 (0.12-1.57)	N/A	Very low
	Social support	2	30	34	−0.38 (−1.12 to 0.35)	53	Very low
**Information or education plus professional psychosocial support plus monitoring**						
	Self-efficacy or mastery	1	17	15	0.17 (−0.52 to 0.87)	N/A	Very low

^a^Grading of recommendations assessment, development, and evaluation system quality assessment.

^b^N/A: not applicable.

For studies that examined *information or education plus combined peer and professional psychosocial support*, results showed a mean increase of 0.85 points (95% CI 0.12 to 1.57) for self-esteem and 0.52 points (95% CI 0.10 to 0.94) for self-efficacy or mastery compared with no or minimal Web-based intervention; the quality of this evidence was very low. For the outcomes of burden, life satisfaction, and social support, there were no statistically significant differences between groups. Finally, the single study that examined *information or education plus professional psychosocial support plus electronic monitoring* found no statistically significant difference between groups for the outcome of self-efficacy or mastery; the quality of this evidence was very low.

## Discussion

### Principal Findings

To the best our knowledge, this paper and its companion paper, focused on caregiver mental health outcomes [21], are the first meta-analyses examining the effect of caregiver-focused, Web-based interventions on outcomes of caregivers of adults with chronic conditions living in the community. This systematic review and meta-analysis showed small to medium beneficial effects of Web-based interventions on caregiver outcomes of self-esteem, self-efficacy, or mastery and strain but no effect on the burden, life satisfaction, reaction to problem behavior, and social support. For Web-based information or education interventions, there was a small effect size on self-efficacy or mastery and strain with a moderate quality of evidence. For Web-based information or education plus professional psychosocial support (1 study), there was a large effect size for self-efficacy or mastery, but the quality of evidence was very low. For Web-based information or education plus combined peer and professional psychosocial support, there was a large effect size for self-esteem and moderate effect size for self-efficacy or mastery, but the quality of the evidence was also very low. Finally, for Web-based information or education plus professional psychosocial support plus electronic monitoring, there was no effect on self-efficacy or mastery.

There are a number of possible reasons why consistent findings across caregiver outcomes were not shown. According to the GRADE scores, the quality of evidence was low or very low for most of the outcomes examined, and none of the outcomes was rated as having high-quality evidence. Furthermore, some outcomes were assessed in only a single study; there was variability in the assessment tools used to assess outcomes, caregiver characteristics varied across studies, and very few studies examined different types of Web-based interventions, reflecting that this is an emerging area of research.

In relation to RoB, 4 studies had high RoB in the area of blinding participants or providers, 3 studies had high RoB in the area of incomplete outcome data, and 2 studies had high RoB for allocation concealment, blinding of outcome assessment, and sequence generation. In addition, there were many areas where RoB could not be determined because of the lack of information in the published papers; for example, RoB was unclear in 12 of 14 studies related to the allocation concealment, in 10 of 14 studies related to blinding of the outcome assessment, and in 9 of 14 studies related to blinding of participants and providers. It is vital that the authors of such trials provide more detailed information about trial procedures using the Consolidated Standards of Reporting Trials guidelines for nonpharmacological interventions [48] because this would enable more accurate assessment of studies for bias and may help to improve the quality of evidence in this area.

The improvements in caregiver self-efficacy or mastery as a result of Web-based interventions in this study are promising. These improvements were observed in Web-based interventions that included information or education in combination with either professional psychosocial support or both peer and professional psychosocial support. Caregiver self-efficacy, or a person’s perception of their ability to perform tasks related to caregiving competently, is a modifiable factor that is important in understanding the effect of Web-based caregiver interventions. Previous research has shown that higher self-efficacy is associated with fewer depressive symptoms among dementia family caregivers [49,50]. Moreover, research suggests that self-efficacy for managing dementia may protect caregivers against burden and depression [51] and that it plays a mediating role between social support and depressive symptoms [52].

For the outcome of self-efficacy or mastery, the addition of professional psychosocial support to information or education only resulted in an increase in SMD from 0.31 to 1.20, whereas the addition of both peer and professional psychosocial support resulted in an increase in SMD from 0.31 to 0.52; this suggests that human support (either professional or peer or a combination of these), as previously shown in the review by Guay et al [13], plays an important role in improving caregiver outcomes.

The companion paper [21], a meta-analysis of the effect of internet-based interventions on caregiver mental health, showed that such interventions also result in a reduction in depressive symptoms, stress or distress, and anxiety. Because the companion paper included many of the same studies in this meta-analysis, it had similar limitations in relation to the quality of the evidence.

A theoretical basis for Web-based interventions has been shown to be effective [13,53]. Half of the included studies reported using theories, such as cognitive theories of stress, to develop their interventions [34,41,43]. Interventions that included behavioral change techniques, such as stress management, may have contributed to significant findings. It is recommended that Web-based interventions for caregivers include a strong theoretical base [54] and include strategies to support improved self-efficacy, stress management, and coping.

### Strengths and Limitations

This review uses meta-analysis to summarize the most relevant trial evidence available on the effects of Web-based interventions on caregiving outcomes. Another strength of this review is the *a priori* selection of rigorous methodological designs, including only RCTs and CCTs. The review was conducted using a comprehensive search strategy and methodologically rigorous processes for systematic reviews and meta-analyses. The included papers were published between 2005 and 2016 with 12 of 14 published since 2012, which reflects the growing interest in Web-based technology to support caregivers. One of the limitations of the review involved the overall low quality of the studies included, despite being RCTs and CCTs. Owing to the considerable heterogeneity of interventions across studies, results were examined according to the types of Web-based interventions. However, there were very few studies that used each type of Web-based intervention across the outcomes of interest.

### Conclusions

This paper and its companion paper [21] are the first meta-analyses of the effect of Web-based interventions for caregivers of community-living adults with chronic conditions on caregiver outcomes. The findings indicate that there is accumulating evidence for the positive effect of caregiver-focused, Web-based interventions to support family and friend caregivers. However, future high-quality research with stronger study designs, larger sample sizes, and the use of standardized tools to facilitate meta-analysis and assessment of clinical relevance are needed to understand the effect of such interventions, particularly multicomponent interventions using peer or professional support.

## Appendix

Multimedia Appendix 1 Search terms.

Multimedia Appendix 2 Detailed characteristics of included studies.

Multimedia Appendix 3 Caregiver outcomes and measurement assessment tools.

Multimedia Appendix 4 Meta-analysis and forest plots.

Multimedia Appendix 5 GRADE assessment details.

## Abbreviations

CCT: controlled clinical trial
GRADE: Grading of Recommendations Assessment, Development, and Evaluation system
RCT: randomized controlled trial
RoB: risk of bias
SMD: standardized mean difference
