# Supplementary material for: Caregiver-Focused, Web-Based Interventions: Systematic Review and Meta-Analysis (Part 2)
Source: J Med Internet Res. 2018 Oct 26;20(10):e11247. doi: 10.2196/11247 (PMC6229518; doi:10.2196/11247)
Supplement: Multimedia Appendix 2 [file jmir_v20i10e11247_app2.pdf]

Multimedia Appendix 2: Detailed Characteristics of Included Studies
Risk of Bias: Low (L); High (H); Unclear (U)

**Study/Location**	**Beauchamp et al, 2005, US [43]**
**Purpose**	Our goal was to evaluate the efficacy of a multimedia support program delivered over the Internet to employed family caregivers of persons with dementia
**Methods**	Design: RCT<br>Setting/ Recruitment Methods: We recruited participants through a national campaign that included wire service advertising, contact notices on corporate Web sites, e-mail announcements on caregiver-related listserves, newsletter articles, and worksite promotional flyers. Interested persons were directed to a Web site that described the study and provided an online screening survey.<br>Inclusion criteria: To allow for the broadest representation of caregivers in the workforce, we required participants to be employed at least part time and have at least four contacts a month caring for a family member with substantial memory problems.
**Participants**	Recruited Sample: 299<br>Baseline Sample: I = 150; C = 149<br>Loss to follow-up: 8 overall (307 participated through the 30-day follow-up assessment, 299 had complete data on all measures)<br>Mean age (years): Overall = 46.9 (12.2)<br>Gender [Male n (%)]: 27%<br>Relationship of caregiver to care recipient: cared for a parent (67%), a spouse or partner (7%), some other relative (23%), or a nonrelative (3%)<br>Ethnicity: Eighty percent of evaluation participants were Caucasian, 4% African American, 8% Hispanic, and 8% other<br>SES status: Ninety percent had completed at least some college or trade school

**Intervention**	Description of Intervention: Caregiver's Friend: Dealing With Dementia is a Web-based multimedia intervention that provides text material and videos that model positive caregiving strategies. Funded by the National Institute on Aging, program content was created for this project by research scientists and instructional designers on the basis of an extensive literature review; interviews with academic gerontologists, social workers, nurses; and focus groups with an ethnically and geographically diverse range of family caregivers.<br>Description of Control: A usual care waitlist control group with no attention-placebo. We informed participants in the control condition by e-mail that they would receive an e-mail in 30 days inviting them to answer another set of questions (the 30-day, T2 follow-up), at which time they would be free to view Caregiver's Friend.<br>Duration of Intervention: 30 days<br>Length of follow-up: 30 days
**Risk of Bias**<br>**Overall: U**	Selection: (U) Insufficient information to make assessment<br>Allocation Concealment: (U) Insufficient information to make assessment<br>Blinding Participants: (U) Insufficient information to make assessment<br>Blinding Assessors: (U) Insufficient information to make assessment<br>Incomplete Data: (L) Drop-out rates =<10%<br>Selective Reporting: (L) Outcomes in methods and results<br>Other: (L) No baseline imbalance

**Study/Location**	**Cristancho-Lacroix et al, 2015, France [34]**
**Purpose**	This study aimed to evaluate through a pilot unblinded randomized controlled trial the efficacy and acceptability of a Web-based psychoeducational program for informal caregivers of persons with Alzheimer's disease (PWAD) based on a mixed methods research design
**Methods**	Design: RCT<br>Setting/Recruitment Methods: The recruitment strategy included flyers and posters placed in the hospital. During the consultations, geriatricians proposed this protocol to caregivers of PWAD. The caregivers interested in the study filled out a contact form. Then a psychologist provided them with the information form, confirmed inclusion criteria, and collected the signed informed consent.

	Inclusion criteria: Eligible participants were required to be French-speaking caregivers of community-dwelling PWAD who met the criteria of the Diagnostic and Statistical Manual of Mental Disorders, 4th Edition. Caregivers had to spend at least 4 hours per week with their relative, be aged 18 years or older, scored 12 or more on the Perceived Stress Scale (PSS-14), and to have access to a computer with Internet connection. Professional caregivers were ineligible.
**Participants**	Recruited Sample: 49<br><br>Baseline Sample: I = 25; C = 24<br><br>Loss to follow-up: I = 3; C = 3<br><br>Mean age (years): Overall, I = 64.2 (10.3); C = 59.0 (12.4)<br><br>Gender [Male n (%)]: I = 36%; C = 33%<br><br>Relationship of caregiver to care recipient: Children (I = 64%; C = 67%) and spouses<br><br>Ethnicity: NR<br><br>SES status: High Level of Education: I=76%, C=75%; Middle level of education: I=24%, C=12%.
**Intervention**	Description of Intervention: The experimental group participants received at baseline a 10-minute training session on how to use the website, a log-in and password, a printed version of the user's manual, and a notebook to write personal ideas about their application of the program's content. Each week, participants had to read through an entire thematic session and fill out a printed satisfaction questionnaire. Other website sections (e.g., relaxation training, forum) were available but not mandatory to complete the program. No modification regarding methodology, program content (except for forum discussions), or the website was done during the course of the study.<br><br>Description of Control: The control and experimental group participants received usual care, in which they were provided with information about the illness during their semiannual follow-up with their geriatrician. The control group participants were given access to the Diapason program at the end of their participation. All participants were advised to look for additional help if necessary and were asked to inform the researcher about it.<br><br>Duration of Intervention: 3 months (12 weekly online sessions)<br><br>Length of follow-up: 3 months post intervention.

**Risk of Bias**<br>**Overall: (H)**	Selection: (L) Computerized random-number/block randomization<br>Allocation Concealment: (U) Insufficient information for assessment<br>Blinding Participants: (H) No blinding<br>Blinding Assessors: (H) No blinding<br>Incomplete Data: (L) No concerns<br>Selective Reporting: (L) No concerns<br>Other: (H) Baseline imbalance between groups

**Study/Location**	**DuBenske et al, 2014 [37] Gustafson et al, 2013, USA [46]**
**Purpose**	In this study, the authors examined the effectiveness of an online support system (Comprehensive Health Enhancement Support System) versus the Internet in relieving physical symptom distress in patients with non–small cell lung cancer (NSCLC).
**Methods**	Design: RCT<br>Setting/ Recruitment Methods: Dyads were identified by their oncologist and were invited to participate by the enrollment coordinator at each site.<br>Inclusion criteria: Eligible dyads consisted of English-speaking adult patients with NSCLC at stage IIIA, IIIB, or IV and a patient-identified primary caregiver who was willing to participate in the study. Patients had to have a clinician-perceived life expectancy of at least 4 months; if patients had brain metastasis, then it had to be stable. Caregivers provided instrumental, emotional, and/or financial support for the patient and were aged at least 18 years.
**Participants**	Recruited Sample: 285<br>Baseline Sample: I = 144; C = 141<br>Loss to follow-up: I = 49; C = 51<br>Mean age (years): Overall: I = 54.57 (12.21); C = 56.56 (12.86)<br>Gender [Male n (%)]: I = 29.5%; C = 33.9%<br>Relationship of caregiver to care recipient: Spouse/partner 72%<br>Ethnicity: NR<br>SES status: Highest level of education high school or less: I = 24 (19.9%), C = 27 (22.5%); Some college courses: I = 28 (23.1), C= 34 (28.3); Advanced degree: I = 69 (57), C=59 (49.2)

**Intervention**	Description of Intervention: Those in the CHESS arm received password-protected access to the CHESS Coping with Lung Cancer website. CHESS was designed to be easy to use and: 1) to provide well organized lung cancer, caregiving, and bereavement information; 2) to serve as a channel for communication with and support from peers, experts, clinicians, and users' social networks; 3) to act as a coach by gathering information from users and providing feedback based on algorithms (decision rules); and 4) to provide tools (e.g., a program to organize support from family and friends) to improve the caregiving experience. CHESS included a clinician report that summarized caregiver and patient ratings of the patient's health status and listed their questions for the next clinic visit. Clinicians received e-mail alerts before a scheduled visit and whenever a symptom rating exceeded 7 on a scale from 0 to 10. CHESS services are described in more detail elsewhere.<br>Description of Control: Participants in the Internet arm received training on using the Internet and a list of Internet sites about lung cancer<br>Duration of Intervention: 6 months (but access for up to 2 years)<br>Length of follow-up: 6 months
**Risk of Bias**<br>**Overall: (H)**	Selection: (U) participants are assigned by random numbers but authors do not indicate if the sequence was computer generated<br>Allocation Concealment: (U) Insufficient information for assessment<br>Blinding Participants: (H) Study non-blinded<br>Blinding Assessors: (H) Study non-blinded<br>Incomplete Data: (H) High attrition<br>Selective Reporting: (L) No concerns<br>Other: (H) Baseline imbalance

**Study/Location**	**Hattink et al, 2015, the Netherlands [35]**
**Purpose**	The objective of the current study was to evaluate the user friendliness, usefulness, and impact of STAR with informal caregivers, volunteers and professional caregivers

**Methods**	Design: RCT<br>Setting/Recruitment Methods: Participants in the Netherlands were recruited through meeting centers for people with dementia and their caregivers, regional branches of the national Alzheimer's organizations, case managers, care organizations, and via announcements through several informative websites targeted at informal caregivers, volunteers, and those with an interest in dementia. In the United Kingdom, participants were recruited through caregivers' cafes, church groups, university service users and caregiver groups, and local dementia care and welfare organization<br>Inclusion criteria: Participants were caring for someone with dementia as an informal caregiver, a volunteer in dementia care, or a professional caregiver, and were living in either the Netherlands or in the United Kingdom
**Participants**	Recruited Sample: 142<br>Baseline Sample: I = 27; C = 32 (informal caregivers)<br>Loss to follow-up: NR<br>Mean age (years): Overall, I = 52.9 (11.43); C = 54.69 (14.36)<br>Gender [Male n (%)]: I = 26.0%; C = 31.0%<br>Relationship of caregiver to care recipient: Partner; child; sibling<br>Ethnicity: NR<br>SES status: NR
**Intervention**	Description of Intervention: The STAR platform was designed to be accessible through any Internet-enabled device so users could access the course at any time and place of their convenience. The STAR training portal consists of an online course with 8 modules relating to different topics. These topics were selected to cover a wide range of topics relating to dementia and dementia care. The modules consist of text, videos, interactive exercises, knowledge tests, and also include references to other websites, literature, and videos<br>Description of Control: People in the control group were informed that they were assigned to the group that could follow the course free of charge after post-test measurements 4 months later<br>Duration of Intervention: 2-4 months<br>Length of follow-up: immediate post (2-4 months)
**Risk of Bias**<br>**Overall: (L)**	Selection: (L) Computer Generated<br>Allocation Concealment: (U) Insufficient information for assessment

	Blinding Participants: (L) No concerns<br>Blinding Assessors: (L) No concerns<br>Incomplete Data: (L) No concerns<br>Selective Reporting: (L) No concerns<br>Other: (L) No concerns

**Study/Location**	**Hattink et al, 2016, the Netherlands, Germany and Belgium [45]**
**Purpose**	This research aimed to integrate three previously developed assistive technology (AT) systems into one modular, multifunctional system, which can support people with dementia carers throughout the course of dementia
**Methods**	Design: RCT (Germany) and pre-test–post-test control group design with matched groups (Netherlands and Belgium)<br>Setting/Recruitment Methods: Care organizations in the three countries via letters (additional information meeting in Germany). Those who consented to participated where then contacted for in-home baseline assessments.<br>Inclusion criteria: participants with MCI or dementia living in the community and their informal carers. Other general inclusion characteristics included a maximum of seven rooms in a one story-house with a maximum size of 180 square meters and a maximum of five exterior doors. The house had to have the possibility for installation of wireless broad-band internet access (if not available already), since all signals of the Rosetta system were transmitted wirelessly and uploaded through an internet server.
**Participants**	Recruited Sample: 42 persons with either mild cognitive impairment (MCI) or dementia (19 in the Netherlands, 11 with MCI in Germany and 12 in Belgium), and 32 of their informal carers.<br>Baseline Sample: Intervention = 5 in Germany 15 (combined) in Netherlands and Belgium; Control = 6 in Germany, 16 (combined) in Netherlands and Belgium<br>Loss to follow-up: I = 9; C = 9<br>Mean age (years): Overall, I = 66; C = 69<br>Gender [Male n (%)]: I = 7/17 (41.2%), C = 6/15 (40.0%)<br>Relationship of caregiver to care recipient: Most carers were either

	partners or children of the person with dementia.<br>Ethnicity: NR<br>SES status: NR
**Intervention**	Description of Intervention: The Rosetta system was installed (sensors, cameras, video home terminal and/or a mobile device) in the homes of the persons with MCI or dementia in the experimental group. After installation, participants received a training explaining how the system works. The intervention consisted of three subsystems: 1. Elderly Day Navigator, including memory support and a Help function on the mobile device to allow caregivers to help persons with dementia find their way home if lost; 2. Early Detection System which allowed electronic monitoring, through sensors, of the person's daily activities shared with caregivers and generating warnings for caregivers if there were significant changes in the patterns of day-to-day living of the person with dementia; and 3. Unattended Autonomous Surveillance system which monitors the person with dementia through sensors and cameras, and detects emergency situations such as falls; Rosetta data was transmitted through the internet. The effective usage period varied from half a month (which was the case for one participant, recruited as replacement for a drop-out) to eight months. Average use was nearly four months<br>Description of Control: Persons in the control group received care and support as usual. This usual care generally consisted of home care for household chores and/or personal care and day care. Some participants received extra care, for example, help with food preparation or visits to the general practitioner.<br>Duration of Intervention: 2 weeks to 8 months<br>Length of follow-up: NR
**Risk of Bias**<br>**Overall: (H)**	Selection: (H) Initially designed as RCT for all three regions (Germany, Netherlands, Belgium), however RCT only maintained in Germany.<br>Allocation Concealment: (H) Pre/post-test control group design with matched groups.<br>Blinding Participants: (U) Insufficient information for assessment<br>Blinding Assessors: (U) Insufficient information for assessment<br>Incomplete Data: (L) No concerns<br>Selective Reporting: (L) No concerns<br>Other: (L) No concerns

**Study/Locatio**	**Kajiyama et al, 2013, US [38]**

**n**	
**Purpose**	Determine if the online iCare Stress Management e-Training Program reduces stress, bother, depression, and poor life quality for dementia family caregivers (CGs)
**Methods**	Design: RCT<br><br>Setting/ Recruitment Methods: CGs were recruited from the community through notices placed in family service agencies and other information and referral resources. Interested CGs contacted research personnel at Photozig, Inc via email or telephone. Following the initial contact all communications with interested persons occurred online using questionnaires<br><br>Inclusion criteria: Screening was a two-step process. In the initial screening, we asked if: 1) they were at least 21 years of age or older; 2) they were caring for an individual with a clinical diagnosis of some type of dementia; and 3) they had access to the Internet on any type of computer or had access to a DVD player. If they answered 'yes' to these questions, they were asked to read the consent form and return a signed copy (either e-mail or regular mail) to indicate willingness to be in the study.
**Participants**	Recruited Sample: 150<br><br>Baseline Sample: Intervention = 75; Control = 75<br><br>Loss to follow-up: I n=25, C n=18<br><br>Mean age: Overall = NR, I= 55.22(11.31) C= 57.02(12.53)<br><br>Gender [Male n (%)]: I: 8(14%), C: 8(17%<br><br>Relationship of caregiver to care recipient: Spouse/Partner: I = 26 (56%), C = 29 (51%); Child: I = 15 (33%), C = 21 (37%); Other relative: I = 2 (4%), C= 4 (7%); Non-relative I = 3 (7%), C = 3 (5%)<br><br>Ethnicity: Caucasian; I = 41 (89%); C = 48 (84%)<br><br>SES status: High school Ed: I = 7 (15), C=12 (21); College: I = 26 (57), C = 26 (46); Graduate: I = 13 (28), C = 19 (33)
**Intervention**	Description of Intervention: iCare (ICC). In the first year of this project, extensive interviews were conducted with caregivers and professionals in the field to develop and test each of the six modules included in the final web-based program. Table 3 provides a brief outline of the Introduction, the modules, and the summary of future actions included in the intervention. The format for completing the ICC was configured so that the modules had to be completed in the order listed in Table 3. There were no minimum time constraints for completing a module built into the program, but participants were encouraged to practice specific assignments in each module over a 7- to 10-day interval before moving to the next one. The iCare program begins with

	an information segment about what 'dementia' means and what are common problems associated with it. Then, there are components on dealing with stress including techniques for relaxation, stress management, and challenging unduly negative thoughts about caregiving; behavioral activation (increasing everyday positive activities for oneself and the PWD); communication skills to improve help-seeking with family and community institutions as well as improving ability to relate to the PWD; managing difficult behaviors of the PWD; and finally, a review of 'healthy habits' (nutrition and exercise) for the CG along with information on national resources they can consult for further on-going assistance<br><br>Description of Control: CGs assigned to the EOC were exposed to a website containing the similar navigational features, but the content focused on information about dementia, obtained from reputable national sources such as the ADEAR program of the National Institute on Aging and the national website of the Alzheimer's Association. In addition, links to certain video-taped information were provided (e.g., the Alzheimer's Project, developed by HBO in collaboration with the National Institute on Aging and the Alzheimer's Association) (The Alzheimer's Project, 2009). Written materials from various health agencies were also provided in a booklet format.<br><br>Duration of Intervention: 3 months<br><br>Length of follow-up: No additional follow ups were completed
**Risk of Bias**<br><br>**Overall: (U)**	Selection: (U) Insufficient information for assessment<br><br>Allocation Concealment: (U) Insufficient information for assessment<br><br>Blinding Participants: (U) Insufficient information for assessment<br><br>Blinding Assessors: (U) Insufficient information for assessment<br><br>Incomplete Data: (H) High attrition with only completers analyses<br><br>Selective Reporting: (L) No concerns<br><br>Other: (L) No concerns

**Study/Location**	**Kim et al, 2013, South Korea [39]**
**Purpose**	The effectiveness of methods to prevent stroke recurrence and of education focusing on learners' needs has not been fully explored. The aims of this study were to assess the effects of such interventions among stroke patients and their primary caregivers and to evaluate the feasibility of a web-based stroke education program

**Methods**	Design: RCT<br><br>Setting /Recruitment Methods: The study was performed in a neurology clinic in Cheonan, South Korea. Individuals who visited a clinic for stroke treatment and had received a clinical diagnosis of ischemic stroke within 12 months post stroke were invited to participate in the study<br><br>Inclusion criteria: Individuals who visited a clinic for stroke treatment and had received a clinical diagnosis of ischemic stroke within 12 months post stroke were invited to participate in the study. The eligibility criteria were as follows: (i) normal cognitive function (a score over 19 on the Korean version of the Mini-Mental State Examination, MMSE-K), (ii) living at home and (iii) Internet access and access to a usable computer
**Participants**	Recruited Sample: 36 caregivers<br><br>Baseline Sample: I =18; C =18<br><br>Loss to follow-up: I = 2; C = 4<br><br>Mean age: overall =53.0 (13.7); I = 49.8 (14.8); C = 57.3 (11.5)<br><br>Gender [Male n (%)]: For stroke patients; I = 13(72.5%), C = 55.6(10%). For caregiver NR.<br><br>Relationship of caregiver to care recipient: I: 66.7% Spouse, 22.2% Son/Daughter, 11.1% Hired help; C: 77.8% Spouse; 22.2% Son/Daughter<br><br>Ethnicity: NR<br><br>SES status: Caregiver above middle school graduate; I = 14(77.8%),; C = 17(94.4%).
**Intervention**	Description of Intervention: The web-based program had four major functions: (i) repeatable playing of video lectures containing tips from health professionals about recurrence prevention and automated quizzes about them; (ii) automatic feedback with regard to the lectures according to the patients' own ratings of their health behaviors; (iii) the ability to join an e-mail service to network with health professionals and (iv) reliable external links to websites containing stroke-related information. Study contents for the nine sessions were selected according to three topical areas: understanding of stroke, recurrence prevention and family life. For the education component, the rehabilitation physician, physical therapist and professor of nursing participated in producing video lectures lasting 15–20 min, using an authoring tool to post Microsoft PowerPoint files. The sessions were designed to be completed on a weekly basis, and they were introduced to participants once per week for a total of 9 weeks. Quiz items were administered after each session on the web; automated feedback was provided to users in response to their inputs. The participants were also able to access external links that contained reliable information on stroke.

	Description of Control: The control group received standard care as prescribed by their physicians and no further recurrence prevention advice; they were contacted for endpoint data collection 3 months after the initial interview.<br>Duration of Intervention: 9 weeks<br>Length of follow-up: 3 months post baseline
**Risk of Bias**<br>**Overall: (U)**	Selection: (L) Computer-generated<br>Allocation Concealment: (U) Insufficient information for assessment<br>Blinding Participants: (H) Blinding of participants not possible<br>Blinding Assessors: (U) Insufficient information for assessment<br>Incomplete Data: (L) <30% dropout and reasons provided<br>Selective Reporting: (L) No concerns<br>Other: (L) No concerns

**Study/Location**	**McLaughlin et al, 2013, US [40]**
**Purpose**	To examine the efficacy of an interactive multimedia intervention that teaches advocacy skills to people caring for a family member with traumatic brain injury
**Methods**	Design: RCT<br>Setting/Recruitment Methods: Participants were recruited through advertising by the Brain Injury Association of America (BIAUSA)<br>Inclusion criteria: Criteria for participation included (a) family member of an adult with a brain injury; (b) providing at least limited support (ie, checking in occasionally, helping with some activities); (c) English speaking; and (d) access to a high-speed Internet connection
**Participants**	Recruited Sample: 201<br>Baseline Sample: I = 104; C = 97<br>Loss to follow-up: I = 14%; C = 6%<br>Mean age: 18-35 years: I = 15 (14.4%); C=9(9.3%)<br>36-50 years: I = 44 (42.3%); C = 51(52.6%)<br>51-60 years: I = 36 (34.6%); C = 29 (29.9%)

	Over 61 years: I = 8 (7.8%); C = 7 (7.2%).<br>Gender [Male n (%)]:I = 5 (14.4%); C=13(13.4%)<br>Relationship of caregiver to care recipient: Family member<br>Ethnicity: I: 86.5% Caucasian; C: 87.6% Caucasian<br>SES status: <$20 000: Control= 14 (14.4); Intervention= 10 (9.6)<br>$20 000-$39 999: Control= 20 (20.6); Intervention= 25 (24.0)<br>$40 000-$59 999: Control= 22 (22.7); Intervention= 17 (16.3)<br>$60 000-$79 999: Control= 19 (19.6); Intervention= 25 (24.0)<br>>$80 000: Control= 20 (20.6); Intervention= 24 (23.1)<br>Did not respond: Control= 2 (2.1); Intervention= 3 (2.9)
**Intervention**	Description of Intervention: The BIP Web site was developed to (a) train family members in advocacy skills, particularly the core skills needed for effective communication including active listening and problem solving (e.g. appropriate body language, acknowledging different perspectives); (b) help users find a broad range of services and supports (i.e. through external links and a library of articles on the Web site; (c) provide strategies for reducing stress (e.g. coping with grief, guilt, and burnout through healthy living and stress management and requesting help); and (d) help determine necessary supports (e.g. independent living needs, transition planning). The training uses text, interactive video examples, and video-based skills exercises<br>Description of Control: directed to the BIAUSA Web site (control group). Like the BIP program, the BIAUSA site contains information for caregivers about managing stress, requesting help from friends and family, and obtaining services; however, its main advocacy focus is legislative. Control participants were asked to use the Web site for a minimum of 30 minutes to match the minimum time treatment participants would view the BIP program<br>Duration of Intervention: 3 months and 10 days<br>Length of follow-up: 10 days from baseline (interim outcomes) and immediate post (3 months after T2 data collection)
**Risk of Bias**<br>**Overall: (U)**	Selection: (U) Insufficient information for assessment<br>Allocation Concealment: (U) Insufficient information for assessment<br>Blinding Participants: (U) Insufficient information for assessment<br>Blinding Assessors: (U) Insufficient information for assessment<br>Incomplete Data: (L) <30% dropout and reasons provided<br>Selective Reporting: (L) No concerns<br>Other: (L) No concerns

**Study/Location**	**Fowler et al**, **2016, US [32]**
**Purpose**	To determine whether caregivers who participated in the Virtual Healthcare Neighborhood reported greater improvements in sleep quality, insomnia, and general self-efficacy compared to caregivers who received standard care
**Methods**	Design: RCT<br>Setting/Recruitment Methods: a convenience sample was used (snowball recruitment), most participants were recruited from the referrals received from eight local Alzheimer's Association support groups<br>Inclusion criteria: participants were primary caregivers (providing the majority of daily care) to homebound care recipients (people who are unable to leave the home without assistance of another individual)
**Participants**	Recruited Sample: 28<br>Baseline Sample: I = 15; C = 13<br>Loss to follow-up: 7 total<br>Mean age of caregiver : I = 60 (12.77); C = 67 (12.2)<br>Gender [Male n (%)]: I: 11 (73%); C: 4 (26%)<br>Relationship of caregiver to care recipient: Spouse: I = 4 (27%), C = 9 (69%); Child: I = 6 (40%), C = 3 (23%); Child-in-law: I = 1 (6.7%), C = 1 (8%); Other: I = 4 (27%), C = 0<br>Ethnicity: White: I = 13 (87%), C = 8 (62%); Black: I = 0, C = 3 (23%); Asian/Hispanic/Hawaiian: I = 2 (13%), C = 2 (15%)<br>SES status: Income <$20,000: I = 3, C = 2; $20000 to $59 999: I = 4 (27%), C = 5 (38%); $60000 to $99999: I = 5 (33%), C = 2 (15%); >$100,000: I = 2 (13%), C=3 (23%); Prefer not to answer: I = 1 (7%), C=1(8%).<br>Education: Highschool;I = 4 (27%), C=2(15%); Some college or 4-year degree: I= 8(53%), C=5(38%); Graduate degree: I=3(20%), C=6(46%),<br>% Employed: I: 9(60%); C: 2(15%)

**Intervention**	Description of Intervention: The VHN is an asynchronous website that provides social support through a blog, specific educational material, and the opportunity to ask questions of the interprofessional team participating in the project. The website was monitored daily by the investigators and was password protected. The interprofessional team developed the educational material, which was posted weekly for a total of 16 weeks. Educational materials comprised a brief outline of each weeks' topic, or module, with accompanying information, links to pertinent websites, and relevant YouTube videos. Review questions were presented after each module and participants were given a simple activity related to the week's topic. The website also included a blog, "Ask the Expert" which allowed caregivers to have a one-on-one discussion with investigators. Sleep data were collected from the sleep actigraphy bands.<br>Description of Control: standard care - control group only used the VHN website to upload their data from the actigraphy band to the VHN website (website was password protected)<br>Duration of Intervention: 16 weeks<br>Length of follow-up: immediate post
**Risk of Bias**<br>**Overall: (U)**	Selection: (L) Computer-generated<br>Allocation Concealment: (U) Insufficient information for assessment<br>Blinding Participants: (H) no blinding<br>Blinding Assessors: (U) Insufficient information for assessment<br>Incomplete Data: (L) <30% dropout and reasons provided<br>Selective Reporting: (L) No concerns<br>Other: (L) No concerns

**Study/Location**	**Núùez-Naveira et al, 2016, Denmark, Poland, Spain [33]**
**Purpose**	To test an e-learning platform for informal caregivers to explore the technical and pedagogical specifications, as well as evaluating the impact of its use on the psychological status of the participants.
**Methods**	Design: RCT<br>Setting/Recruitment Methods: Participants were recruited again from different local Alzheimer’s associations of adult day-care centers: from

	the Danish Alzheimer Association (DAA) and the Skanderborg municipality (SKAN) in Denmark, from Poznan, Walcz, Ciechocinek, and Koszalin in Poland, and from the Gerontological Complex La Milagrosa, Saraiva-Mar´ın, and Afal-Ferrolterra in Spain<br><br>Inclusion criteria: The inclusion criteria for the study were as follows: (1) taking care of a person diagnosed with dementia by a specialist or a neurologist, according to the criteria of the Classification of Mental and Behavioural Disorders, 10th revision, or the Diagnostic and Statistical Manual of Mental Disorders, 4th edition, text revision , or the National Institute of Neurological Disorders and Stroke-Alzheimer Disease and Related Disorders Association; (2) being the primary caregiver in the following aspects: executing basic care tasks for a minimum of 6 weeks, receiving no remuneration for caregiving service (except from a few Danish caregivers receiving economical compensation for reducing their ordinary working hours while caring for their demented relative), and devoting much time to patient care; (3) suffering a burden according to the 22-item version of the Zarit Burden Interview, using a cut-off point of 24, which was determined to identify family caregivers who are at risk for depression; and (4) signing the informed consent form to participate in the study. The exclusion criteria were to present some of the following conditions that might prevent the evaluation of the participant or the interaction with the platform: cognitive impairment, illiterate, severe hearing and visual or motoric problems.
**Participants**	Recruited Sample: 77<br><br>Baseline Sample: I = 36; C = 41<br><br>Loss to follow-up: I = 6; C = 10<br><br>Mean age (years): NR<br><br>Gender [Male n (%)]: I = 9 (30.0%); C = 13 (41.9%)<br><br>Relationship of caregiver to care recipient: The most frequent forms of support used were relative and friends (44.3%)<br><br>Ethnicity: NR<br><br>SES status: 46.5% medium level of education
**Intervention**	Description of Intervention: The understAID consists on a Learning section with a database of contents organized in 5 modules with information about 15 different topics. The topics cover information about the care of a person with dementia and caring for oneself as a caregiver. The topics consist of text, videos, and images and they also include references to other websites. The modules and topics included in understAID are Module 1, Cognitive Declines (Topics: Attention, Memory, and Orientation); Module 2, Daily Tasks (Topics: Bathing,

	Incontinence, Massage and Touch, and Physical Exercises); Module 3, Behavioural Changes (Topics: Anxiety and Agitated Behaviour, Depressive Mood, Manic Symptoms, and Emotional Control and Recognition); Module 4, Social Activities (Communication and Apathy and Loss of Motivation); and Module 5, You as a Caregiver (Topics: Coping with Own Stress and Motivation). It also contains a Daily Task section with the option of using a calendar and reminders for appointments and medication intake. Additionally, it has a Social Network section where the caregivers can interact with other participants and exchange information and opinions. This section was moderated by the researchers of the study.<br><br>Description of Control: Participants in the control group did not use the application and maintained their usual lifestyle<br><br>Duration of Intervention: 3 months<br><br>Length of follow-up: immediate post (3 months)
**Risk of Bias**<br><br>**Overall: (U)**	Selection: (L) Computer generated<br><br>Allocation Concealment: (U) Insufficient information for assessment<br><br>Blinding Participants: (U) Insufficient information for assessment<br><br>Blinding Assessors: (U) Insufficient information for assessment<br><br>Incomplete Data: (L) No concerns<br><br>Selective Reporting: (L) No concerns<br><br>Other: (L) No concerns

**Study/Location**	**Pagán-Ortiz et al, 2014, USA [44]**
**Purpose**	To assess the effectiveness of a website developed to increase knowledge of ADRD, caregivers’ self-efficacy for caregiving (competence), perceived social support, and decreasing caregiver burden and emotional distress
**Methods**	Design: CCT<br><br>Setting/Recruitment Methods: “Participants were "recruited from an English as a Second Language class at a community-based organization in

	Boston, Massachusetts. Participants from Puerto Rico and Massachusetts were recruited via outreach strategies that included: letters, press releases, flyers, as well as phone calls to agencies in contact with caregivers. In Mexico, participants were recruited from a pool of caregivers who received social support services at a neurology teaching hospital.<br><br>Inclusion criteria: NR
**Participants**	Recruited Sample: 72<br><br>Baseline Sample: I = 17; C = 23<br><br>Loss to follow-up: I = 2; C = 6<br><br>Mean age (years): Overall, Range 42 to 78<br><br>Gender [Male n (%)]: NR<br><br>Relationship of caregiver to care recipient: NR<br><br>Ethnicity: 100% Spanish speaking<br><br>SES status: NR
**Intervention**	Description of Intervention: The intervention group went through four sessions of approximately 1 to 1-1/2 hr each. The first intervention group session (pretest session) was devoted to providing an overview of the study, familiarizing the caregiver with Cuidate Cuidador, and administering the pretest. The next two intervention group sessions were devoted to ensuring participants' ability to use Cuidate Cuidador's key features. Caregivers who were not computer-literate received specific assistance to navigate through the web site's key component features. The fourth session (posttest session) took place at the 1-month point, and was devoted to the administration of the posttest evaluation, as well as a general debriefing for the study.<br><br>Description of Control: Participants assigned to the control group completed two sessions. A first session was devoted to providing an overview of the study, and administering the pretest. Participants received printed Spanish-language educational materials on Alzheimer's caregiving. The content covered in the printed materials were similar to the topics offered in Cuidate Cuidador, but were obtained from other sources. Participants were instructed to take time between the pretest and posttest to review and use the educational materials as a reference. The second session (posttest session) took place at a 1-month follow-up, and was devoted to the administration of the post-test outcome measures, as well as a debriefing.<br><br>Duration of Intervention: 1 month<br><br>Length of follow-up: immediate post (1 month)

**Risk of Bias**<br>**Overall: (H)**	Selection: (H) Non-randomized<br>Allocation Concealment: (H) Non-randomized<br>Blinding Participants: (U) Insufficient information for assessment<br>Blinding Assessors: (U) Insufficient information for assessment<br>Incomplete Data: (L) No concerns<br>Selective Reporting: (L) No concerns<br>Other: (U) Insufficient information for assessment

**Study/Location**	**Pierce et al, 2009, USA [42]**
**Purpose**	We hypothesised that carers of stroke survivors who participate in the Web-based intervention, Caring Web would have higher well-being than non-Web users. We also postulated that those survivors whose carers participated in Caring Web would use fewer healthcare services.
**Methods**	Design: RCT<br>Setting/ Recruitment Methods: Subjects were recruited from four rehabilitation centres from which first-time stroke survivors were discharged to home in two Midwestern states.<br>Inclusion criteria: For this study, subjects were the primary person (4age 21) responsible for providing day-to-day care for a person with a first-time stroke who had completed treatment and was discharged to home in northern Ohio or southern Michigan. In addition, carers were required to read, write and understand English and have a telephone and television to facilitate MSN TV and Internet access. All potential subjects were novice Internet users. Subjects assigned to either the Web or non-Web user group did not have Internet access in their homes at the time of enrolment into the study. Non-Web users were also told not to purchase or use Internet service during the study but received usual medical care.
**Participants**	Recruited Sample: 144<br>Baseline Sample: I = 51; C = 52<br>Loss to follow-up: 30<br>Mean age (years): Overall: I = 54 (12.2), C = 55 (13.1)<br>Gender [Male n (%)]: I = 11 (30.6%); C = 7 (18.9%)<br>Relationship of caregiver to care recipient: Spouse: I = 42%, C = 51%

	Ethnicity: Caucasian: I=86%, C=84%<br>SES status: Had at least a high school education: I=86%, C=97%
**Intervention**	Description of Intervention: The intervention was constructed with four interrelated components for carers: (1) linked Web sites about stroke and caring; (2) customised educational information or tips specific to carers' needs; (3) an email forum to ask a nurse specialist and a rehabilitation team (therapists, pharmacist, dietitian, social worker and physician) any questions in private and (4) a non-structured email discussion amongst all participants facilitated by the nurse<br>Description of Control: Non-web supported usual care<br>Duration of Intervention: 1 year.<br>Length of follow-up: immediate post (1 year).
**Risk of Bias**<br>**Overall: (U)**	Selection: (U) blocked randomisation scheme with no information on how it was generated.<br>Allocation Concealment: (U) Insufficient information for assessment<br>Blinding Participants: (U) Insufficient information for assessment<br>Blinding Assessors: (U) Insufficient information for assessment<br>Incomplete Data: (H) 30% attrition<br>Selective Reporting: (L) No concerns<br>Other: (L) No concerns

**Study/Location**	**Smith et al, 2012, US [41] Companion Steiner & Pierce, 2002 [47]**
**Purpose**	To develop and test the efficacy of a Web-based intervention for alleviating depression in male stroke survivors (SSs) and their spousal caregivers (CGs) that blends both peer and professional support.
**Methods**	Design: RCT<br>Setting /Recruitment Methods: Dyads were recruited nationally through notices on Web sites and listserv announcements of key organizations (e.g., National Stroke Association; Family Caregiver Alliance).<br>Inclusion criteria: the female CG provided care at home to a husband after stroke; either the SS or CG scored five or more on the PHQ-9 (at least mild depression); neither SS nor CG were medically unstable or terminally ill; and both were cognitively able to participate.

**Participants**	Recruited Sample: 38<br>Baseline Sample: I = 19; C = 19<br>Loss to follow-up: 3<br>Mean age (years): I = 55.3 (6.9); C = 54.9 (12.9)<br>Gender [Male n (%)]: 100% Female<br>Relationship of caregiver to care recipient: wives of care recipient<br>Ethnicity: primarily Caucasian<br>SES status: <$20,000: Intervention = 4 (28.6); Control = 3 (18.8)<br>$21,000–$35,999: Intervention = 6 (42.8); Control = 8 (50.0)<br>$36,000–$50,999: Intervention = 0 (0); Control = 3 (18.8)<br>$60,000: Intervention = 4 (28.6); Control = 2 (12.5)
**Intervention**	Description of Intervention: The intervention consisted of five components designed to provide CGs with knowledge, resources, and skills to help them both reduce their personal distress and to provide optimal emotional care to the SS: Professional Guide, Educational Videos, Online Chat Sessions, E-mail and Message Board, and Resource Room.<br>Description of Control: Those CGs assigned to this condition had access to the Resource Room only. At the RCT outset, they were asked to watch an online video in which the same Professional Guide explained the features of the Resource Room and encouraged CGs to use it as a caregiving resource. There was no further exposure to the Professional Guide beyond that video. A weekly caregiving tip was also presented online, but none overlapped with content covered in the intervention condition. A toll free phone number was provided in case CGs encountered technological problems while accessing the Resource Room, or if a medical emergency occurred. Halfway through the RCT, an assistant phoned CGs to see if they encountered technical difficulties in accessing the Resource Room. Participants in both RCT conditions received identical computer resources for accessing Web-based information and support. The critical difference was that the control condition had no exposure to the key intervention components.<br>Duration of Intervention: 11 weeks<br>Length of follow-up: Immediate post and 1 month follow-up
**Risk of Bias**<br>**Overall: (L)**	Selection: (L) Computer generated<br>Allocation Concealment: (U) Insufficient information for assessment<br>Blinding Participants: (U) Insufficient information for assessment

	Blinding Assessors: (L) Blinded assessors<br>Incomplete Data: (L) No concerns<br>Selective Reporting: (L) No concerns<br>Other: (L) no concerns

**Study/Location**	**Torkamani et al, 2014, UK [36]**
**Purpose**	The current study is a multi-center randomized controlled evaluation of a technology platform specifically designed for PwD living at home and their carers.
**Methods**	Design: RCT<br>Setting/Recruitment Methods: Hospital outpatients identified as having dementia were screened for functional dependency and cognitive impairment using the Barthel Index.<br>Inclusion criteria: Patients living at home with a full time carer, a BI score of at least 35 (indicating some degree of independence), and a MMSE score of at least 9 and no more than 21 (indicating moderate to mild cognitive impairment) were recruited. Patients either had dementia as their primary condition or dementia as part of Parkinson's disease.
**Participants**	Recruited Sample: 60<br>Baseline Sample: I = 30; C = 30<br>Loss to follow-up: NR<br>Mean age (years): Overall, 60.69 (13.09)<br>Gender [Male n (%)]: NR<br>Relationship of caregiver to care recipient: NR<br>Ethnicity: NR<br>SES status: NR
**Intervention**	Description of Intervention: ALADDIN is a computerized platform designed to offer avenues of support and information to the carer. It also manages and communicates information related to the PwD and their carers from their home to the clinicians, facilitating distant monitoring. ALADDIN has four key features: 'ALADDIN TV', 'SOCIAL NETWORKING', 'MY TASKS', and 'CONTACT US'.<br>Description of Control: The participants in the control group were only assessed at the three time points, without any further contact or

	intervention.<br>Duration of Intervention: 6 months<br>Length of follow-up: immediate post (6 months)
**Risk of Bias Overall: (U)**	Selection: (U) Insufficient information for assessment<br>Allocation Concealment: (U) Insufficient information for assessment<br>Blinding Participants: (U) Insufficient information for assessment<br>Blinding Assessors: (U) Insufficient information for assessment<br>Incomplete Data: (L) No concerns<br>Selective Reporting: (L) No concerns<br>Other: (L) No concerns
