# Supplementary material for: Caregiver-Focused, Web-Based Interventions: Systematic Review and Meta-Analysis (Part 2)
Source: J Med Internet Res. 2018 Oct 26;20(10):e11247. doi: 10.2196/11247 (PMC6229518; doi:10.2196/11247)
Supplement: Multimedia Appendix 3 [file jmir_v20i10e11247_app3.pdf]

Multimedia Appendix 3. Caregiver Outcomes and Measurement Assessment Tools

Outcome	Measurement Assessment Tool or Scale
Caregiver Burden	Zarit Burden Interview [34, 44, 36]<br>Caregiver Quality of Life-Cancer Scale Burden Subscale [37]<br>One question source not identified [35]
Life Satisfaction	Satisfaction with Life Scale [42, 40]<br>Revised Caregiving Satisfaction Scale [33]
Self-efficacy or Mastery	Revised Scale for Caregiving Self-Efficacy [34]<br>6 self-efficacy of caregiving items source not reported [43]<br>Short Sense of Competence Questionnaire [35, 45]<br>Personal Mastery Scale (44, 41, 39]<br>General Self-Efficacy Scale [32]<br>Caregiver Competence Scale [33]<br>Caregiving Mastery [39]
Reaction to Problem Behavior	Revised Memory and Behavior Problems Checklist [34, 38]
Self-esteem	Rosenberg Self-Esteem Scale [41]
Caregiver Strain	Caregiver Strain Index [43]
Social Support	Lubben Social Network Scale [44]<br>MOS Social Support Survey [41]
