# Supplementary material for: Caregiver-Focused, Web-Based Interventions: Systematic Review and Meta-Analysis (Part 2)
Source: J Med Internet Res. 2018 Oct 26;20(10):e11247. doi: 10.2196/11247 (PMC6229518; doi:10.2196/11247)

Multimedia Appendix 4: Meta-analysis and forest plots

Change in Caregiver Burden

Study or Subgroup	Experimental Mean	Experimental SD	Experimental Total	Control Mean	Control SD	Control Total	Weight	Std. Mean Difference IV, Random, 95% CI
**3.1.1 Internet: Information/ Education + Psychosocial support - Peer**								
Cristancho-Lacroix, 2015	0.3	6.6	25	-1.5	6.1	24	19.4%	0.28 [-0.28, 0.84]
Hattink, 2015	-0.24	0.749	21	-0.28	0.756	25	18.7%	0.05 [-0.53, 0.63]
**Subtotal (95% CI)**			**46**			**49**	**38.1%**	**0.17 [-0.24, 0.57]**
Heterogeneity: Tau² = 0.00; Chi² = 0.30, df = 1 (P = 0.58); I² = 0%								
Test for overall effect: Z = 0.82 (P = 0.41)								
**3.1.2 Internet: Information/ Education + Psychosocial support - Peers + Psychosocial support - Professional**								
DuBenske, 2014	-4.07	4.496	44	-1.56	5.723	51	26.0%	-0.48 [-0.89, -0.07]
Pagan-Ortiz, 2014	0.09	0.536	15	-0.12	0.442	17	14.9%	0.42 [-0.28, 1.12]
Torkamani, 2014	3.14	15.293	27	1.53	12.199	30	21.0%	0.12 [-0.40, 0.64]
**Subtotal (95% CI)**			**86**			**98**	**61.9%**	**-0.03 [-0.57, 0.50]**
Heterogeneity: Tau² = 0.15; Chi² = 6.00, df = 2 (P = 0.05); I² = 67%								
Test for overall effect: Z = 0.13 (P = 0.90)								
**Total (95% CI)**			**132**			**147**	**100.0%**	**0.03 [-0.31, 0.36]**
Heterogeneity: Tau² = 0.07; Chi² = 7.74, df = 4 (P = 0.10); I² = 48%								
Test for overall effect: Z = 0.15 (P = 0.88)								
Test for subgroup differences: Chi² = 0.35, df = 1 (P = 0.55), I² = 0%								

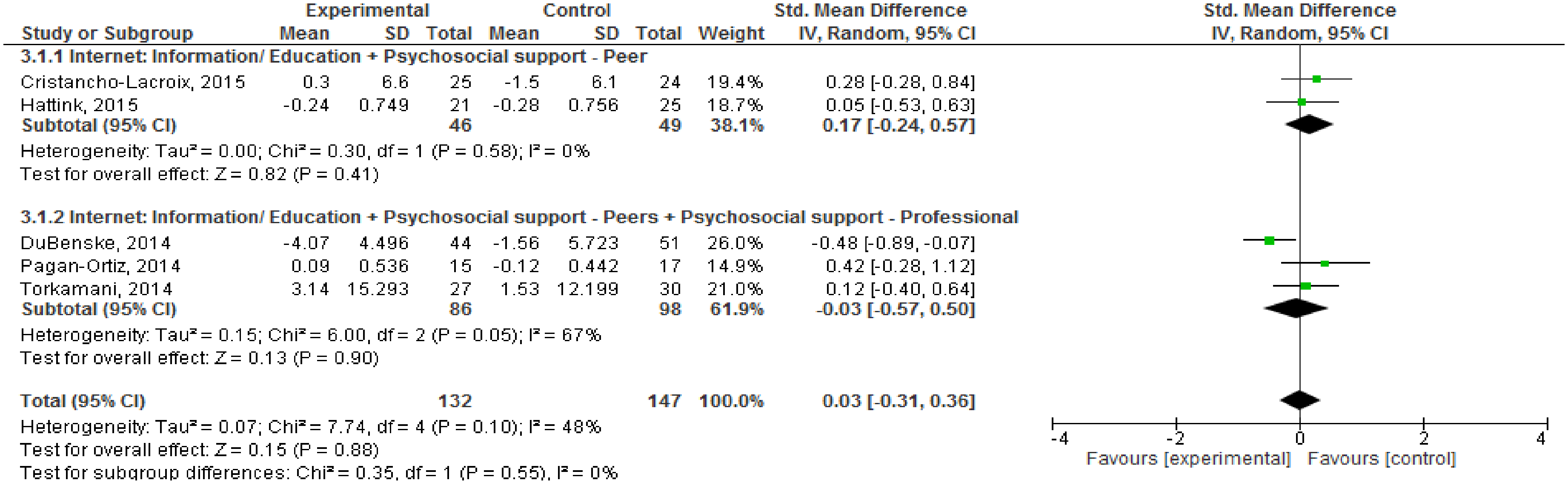

Change in Life Satisfaction

Study or Subgroup	Experimental Mean	Experimental SD	Experimental Total	Control Mean	Control SD	Control Total	Weight	Std. Mean Difference IV, Random, 95% CI
**3.3.1 Internet: Information / Education**								
McLaughlin, 2013	-0.13	1.547	104	0.2	1.418	97	59.9%	-0.22 [-0.50, 0.06]
**Subtotal (95% CI)**			**104**			**97**	**59.9%**	**-0.22 [-0.50, 0.06]**
Heterogeneity: Not applicable								
Test for overall effect: Z = 1.56 (P = 0.12)								
**3.3.2 Internet: Information/ Education + Psychosocial support - Peer**								
Núñez-Naveira, 2016	-0.07	4.318	30	-0.45	5.451	31	18.3%	0.08 [-0.43, 0.58]
**Subtotal (95% CI)**			**30**			**31**	**18.3%**	**0.08 [-0.43, 0.58]**
Heterogeneity: Not applicable								
Test for overall effect: Z = 0.30 (P = 0.77)								
**3.3.3 Internet: Information/ Education + Psychosocial support - Peers + Psychosocial support - Professional**								
Pierce, 2009	-0.6	4.796	36	0.5	4.208	37	21.8%	-0.24 [-0.70, 0.22]
**Subtotal (95% CI)**			**36**			**37**	**21.8%**	**-0.24 [-0.70, 0.22]**
Heterogeneity: Not applicable								
Test for overall effect: Z = 1.03 (P = 0.30)								
**Total (95% CI)**			**170**			**165**	**100.0%**	**-0.17 [-0.39, 0.04]**
Heterogeneity: Tau² = 0.00; Chi² = 1.15, df = 2 (P = 0.56); I² = 0%								
Test for overall effect: Z = 1.56 (P = 0.12)								
Test for subgroup differences: Chi² = 1.15, df = 2 (P = 0.56), I² = 0%								

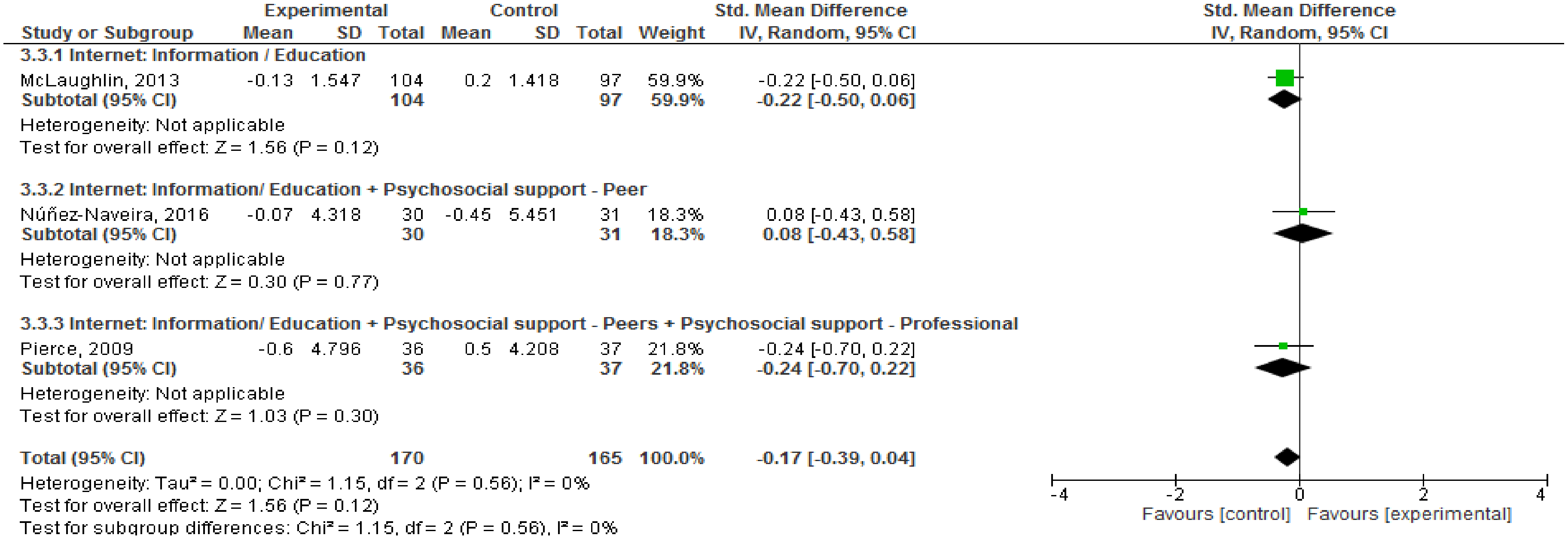

Change in Self-efficacy / Mastery

Study or Subgroup	Experimental Mean	Experimental SD	Experimental Total	Control Mean	Control SD	Control Total	Weight	Std. Mean Difference IV, Random, 95% CI
**3.2.1 Internet: Information / Education**								
Beauchamp, 2005	2.5	5.43	150	0.8	5.66	149	21.9%	0.31 [0.08, 0.53]
**Subtotal (95% CI)**			**150**			**149**	**21.9%**	**0.31 [0.08, 0.53]**
Heterogeneity: Not applicable								
Test for overall effect: Z = 2.63 (P = 0.009)								
**3.2.2 Internet: Information/ Education + Psychosocial support - Peer**								
Cristancho-Lacroix, 2015	-3.2	14.1	25	-0.5	15.5	24	11.6%	-0.18 [-0.74, 0.38]
Hattink, 2015	0.24	0.836	21	-0.5	1.08	25	10.7%	0.74 [0.14, 1.35]
Núñez-Naveira, 2016	-0.23	1.741	30	-0.06	1.744	31	13.1%	-0.10 [-0.60, 0.41]
**Subtotal (95% CI)**			**76**			**80**	**35.3%**	**0.14 [-0.41, 0.69]**
Heterogeneity: Tau² = 0.16; Chi² = 5.95, df = 2 (P = 0.05); I² = 66%								
Test for overall effect: Z = 0.50 (P = 0.62)								
**3.2.3 Internet: Information/ Education + Psychosocial support - Professional**								
Kim, 2013	2.8	1.895	18	0.4	2.017	18	8.6%	1.20 [0.48, 1.92]
**Subtotal (95% CI)**			**18**			**18**	**8.6%**	**1.20 [0.48, 1.92]**
Heterogeneity: Not applicable								
Test for overall effect: Z = 3.28 (P = 0.001)								
**3.2.4 Internet: Information/ Education + Psychosocial support - Peers + Psychosocial support - Professional**								
Nesbitt-Fowler, 2016	-0.2	5.59	15	-4	5.995	13	7.8%	0.64 [-0.13, 1.40]
Pagan-Ortiz, 2014	0.08	0.351	15	-0.06	0.423	17	8.8%	0.35 [-0.35, 1.05]
Smith, 2012	1.2	2.195	15	-0.3	2.698	17	8.6%	0.59 [-0.12, 1.30]
**Subtotal (95% CI)**			**45**			**47**	**25.3%**	**0.52 [0.10, 0.94]**
Heterogeneity: Tau² = 0.00; Chi² = 0.36, df = 2 (P = 0.84); I² = 0%								
Test for overall effect: Z = 2.43 (P = 0.01)								
**3.2.5 Internet + telephone: Monitoring + Psychosocial support – Peers + Psychosocial support - Professional**								
Hattink, 2016	0	1.18	17	-0.17	0.604	15	8.9%	0.17 [-0.52, 0.87]
**Subtotal (95% CI)**			**17**			**15**	**8.9%**	**0.17 [-0.52, 0.87]**
Heterogeneity: Not applicable								
Test for overall effect: Z = 0.49 (P = 0.63)								
**Total (95% CI)**			**306**			**309**	**100.0%**	**0.36 [0.11, 0.62]**
Heterogeneity: Tau² = 0.06; Chi² = 14.79, df = 8 (P = 0.06); I² = 46%								
Test for overall effect: Z = 2.80 (P = 0.005)								
Test for subgroup differences: Chi² = 6.91, df = 4 (P = 0.14), I² = 42.1%								

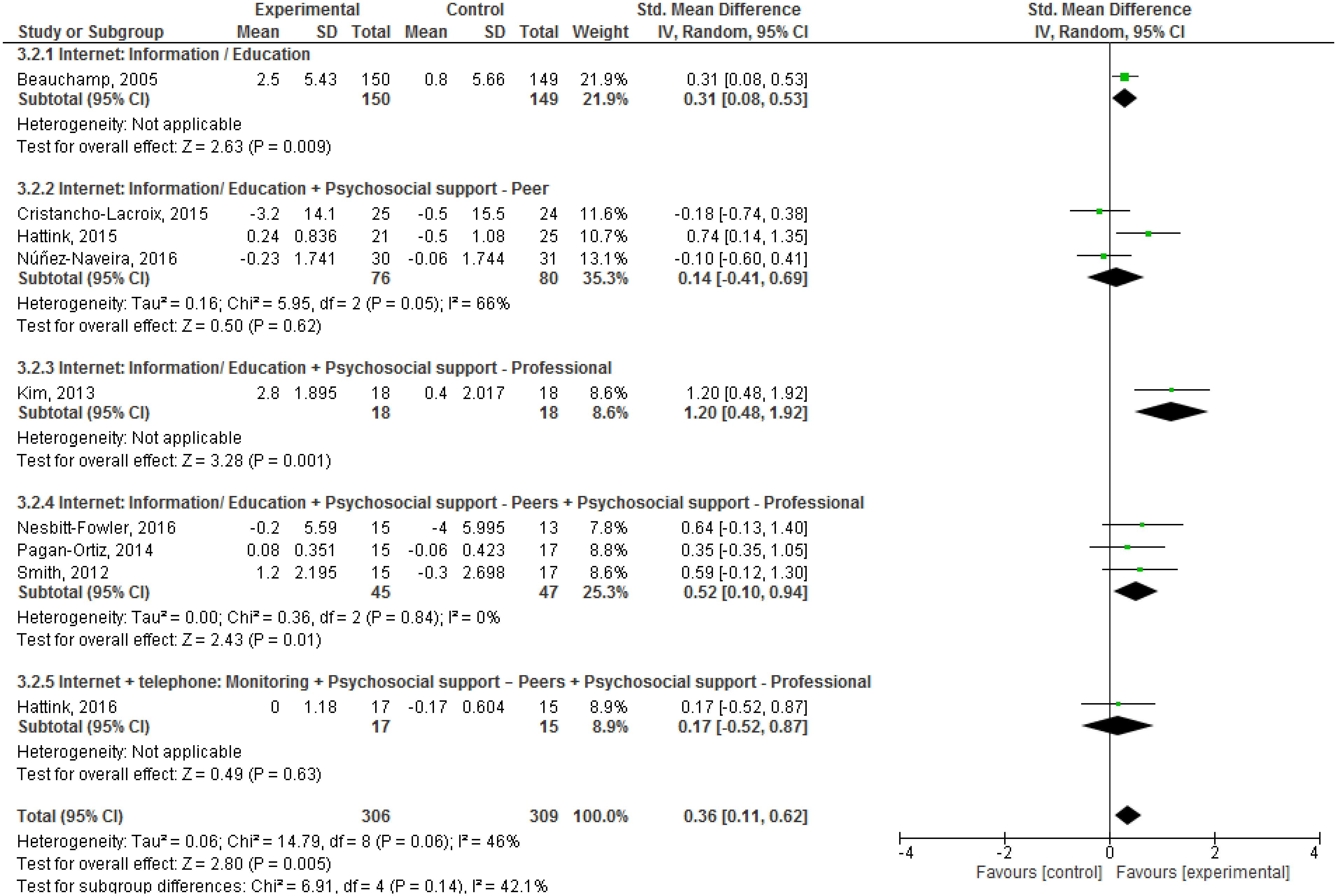

Change in Reaction to Problem Behavior

Study or Subgroup	Experimental Mean	Experimental SD	Experimental Total	Control Mean	Control SD	Control Total	Weight	Std. Mean Difference IV, Random, 95% CI
**1.6.1 Internet: Information / Education**								
Kajiyama, 2013	-0.34	0.593	46	-0.12	0.636	57	56.5%	-0.35 [-0.75, 0.04]
**Subtotal (95% CI)**			**46**			**57**	**56.5%**	**-0.35 [-0.75, 0.04]**
Heterogeneity: Not applicable								
Test for overall effect: Z = 1.77 (P = 0.08)								
**1.6.2 Internet: Information/ Education + Psychosocial support - Peer**								
Cristancho-Lacroix, 2015	0	0.4	25	-0.1	0.5	24	43.5%	0.22 [-0.34, 0.78]
**Subtotal (95% CI)**			**25**			**24**	**43.5%**	**0.22 [-0.34, 0.78]**
Heterogeneity: Not applicable								
Test for overall effect: Z = 0.76 (P = 0.45)								
**Total (95% CI)**			**71**			**81**	**100.0%**	**-0.10 [-0.66, 0.45]**
Heterogeneity: Tau² = 0.10; Chi² = 2.68, df = 1 (P = 0.10); I² = 63%								
Test for overall effect: Z = 0.37 (P = 0.71)								
Test for subgroup differences: Chi² = 2.68, df = 1 (P = 0.10), I² = 62.6%								

Std. Mean Difference
IV, Random, 95% CI

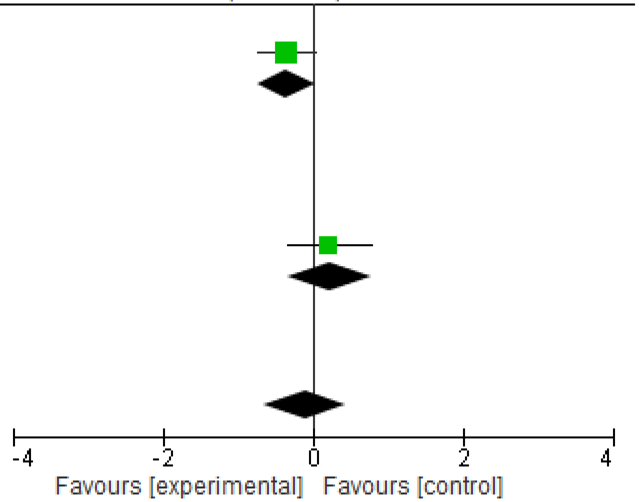

Change in Self-esteem

Study or Subgroup	Experimental Mean	Experimental SD	Experimental Total	Control Mean	Control SD	Control Total	Weight	Std. Mean Difference IV, Random, 95% CI
**3.4.1 Internet: Information/ Education + Psychosocial support - Peers + Psychosocial support - Professional**								
Smith, 2012	2.8	4.526	15	-0.7	3.545	17	100.0%	0.85 [0.12, 1.57]
**Subtotal (95% CI)**			**15**			**17**	**100.0%**	**0.85 [0.12, 1.57]**
Heterogeneity: Not applicable								
Test for overall effect: Z = 2.28 (P = 0.02)								
**Total (95% CI)**			**15**			**17**	**100.0%**	**0.85 [0.12, 1.57]**
Heterogeneity: Not applicable								
Test for overall effect: Z = 2.28 (P = 0.02)								
Test for subgroup differences: Not applicable								

Std. Mean Difference
IV, Random, 95% CI

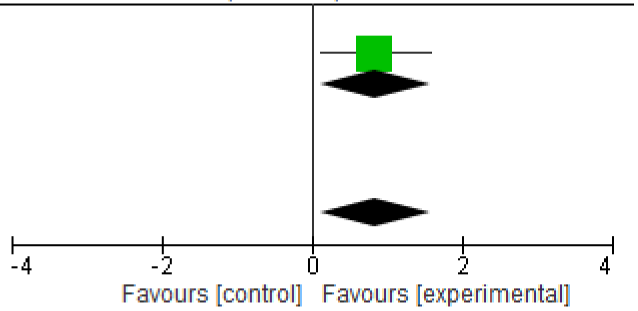

Change in Caregiver Strain

Study or Subgroup	Experimental Mean	Experimental SD	Experimental Total	Control Mean	Control SD	Control Total	Weight	Std. Mean Difference IV, Random, 95% CI
**3.6.1 Internet: Information / Education**								
Beauchamp, 2005	-2.1	7.04	150	0.1	6.859	149	100.0%	-0.32 [-0.54, -0.09]
**Subtotal (95% CI)**			**150**			**149**	**100.0%**	**-0.32 [-0.54, -0.09]**
Heterogeneity: Not applicable								
Test for overall effect: Z = 2.71 (P = 0.007)								
**Total (95% CI)**			**150**			**149**	**100.0%**	**-0.32 [-0.54, -0.09]**
Heterogeneity: Not applicable								
Test for overall effect: Z = 2.71 (P = 0.007)								
Test for subgroup differences: Not applicable								

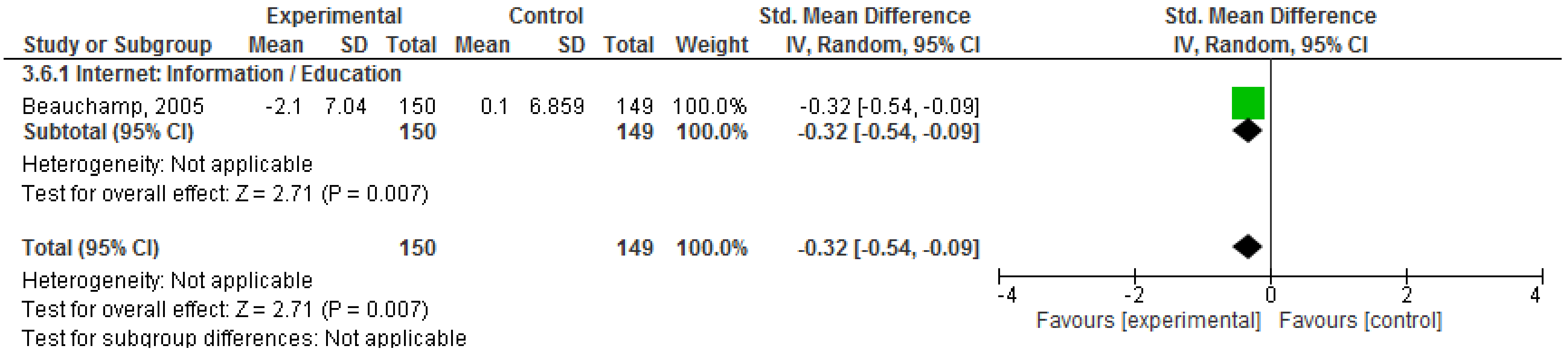

Change in Social Support

Study or Subgroup	Experimental Mean	Experimental SD	Experimental Total	Control Mean	Control SD	Control Total	Weight	Std. Mean Difference IV, Random, 95% CI
**3.5.1 Internet: Information/ Education + Psychosocial support - Peers + Psychosocial support - Professional**								
Pagan-Ortiz, 2014	-0.05	0.444	15	-0.04	0.726	17	50.9%	-0.02 [-0.71, 0.68]
Smith, 2012	1.1	4.594	15	5.1	5.503	17	49.1%	-0.76 [-1.49, -0.04]
**Subtotal (95% CI)**			**30**			**34**	**100.0%**	**-0.38 [-1.12, 0.35]**
Heterogeneity: Tau² = 0.15; Chi² = 2.14, df = 1 (P = 0.14); I² = 53%								
Test for overall effect: Z = 1.02 (P = 0.31)								
**Total (95% CI)**			**30**			**34**	**100.0%**	**-0.38 [-1.12, 0.35]**
Heterogeneity: Tau² = 0.15; Chi² = 2.14, df = 1 (P = 0.14); I² = 53%								
Test for overall effect: Z = 1.02 (P = 0.31)								
Test for subgroup differences: Not applicable								

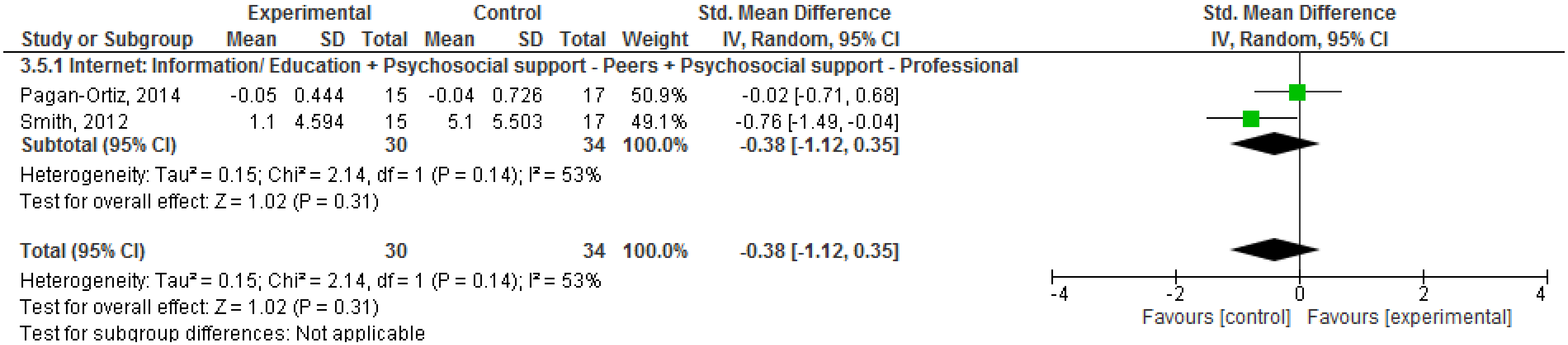

Supplement: Multimedia Appendix 4 [file jmir_v20i10e11247_app4.pdf]
