# Supplementary material for: Caregiver-Focused, Web-Based Interventions: Systematic Review and Meta-Analysis (Part 2)
Source: J Med Internet Res. 2018 Oct 26;20(10):e11247. doi: 10.2196/11247 (PMC6229518; doi:10.2196/11247)
Supplement: Multimedia Appendix 5 [file jmir_v20i10e11247_app5.pdf]

Multimedia Appendix 5. Detailed GRADE Evidence Tables

Table 1: GRADE table for any web-based-based intervention

Patient or population: Caregivers
Intervention: Web-based interventions
Comparison: Control

Outcomes	Anticipated absolute effects* (95% CI)		№ of participants (studies)	Quality of the evidence (GRADE)	Comments
	Risk with Control	Risk with web-based interventions			
Change in Caregiver Burden	-	SMD **0.03 SD higher** (0.31 lower to 0.36 higher)	279 (5 RCTs) [a]	⨁◯◯◯ VERY LOW [b,c,d]	Assessed using 22-item Zarit Burden Interview (range: 0-88) in 3 studies, Caregiver Quality of Life – Cancer Scale (CQOLC) Burden Subscale (10 Likert-type items) in one and 1- item scale in one study.

Multimedia Appendix 5. Detailed GRADE Evidence Tables

Table 1: GRADE table for any web-based-based intervention

Patient or population: Caregivers
Intervention: Web-based interventions
Comparison: Control

Outcomes	**Anticipated absolute effects*** (95% CI)		№ of participants (studies) Quality of the evidence (GRADE)	Comments	
	**Risk with Control**	**Risk with web-based interventions**			
Change in Self-efficacy / Mastery	-	SMD **0.36 SD higher** (0.11 higher to 0.62 higher)	615 (9 RCTs) [e] ⊕⊕◯◯ LOW [b,c]	Assessed using Short Sense of Competence Questionnaire, Revised Scale for Caregiving Self-Efficacy, Pearlin Mastery Scale, Caregiver Competence Scale & General Self-Efficacy scale.	
Change in Life satisfaction	-	SMD **0.17 SD lower** (0.39 lower to 0.04 higher)	335 (3 RCTs) [f]	⊕◯◯◯ VERY LOW [b,c,g]	Assessed using 5-item Satisfaction with Life Scale (range: 1-35) in two studies and 6-item Revised Caregiving Satisfaction Scale (range: 0-30) in one study.

Multimedia Appendix 5. Detailed GRADE Evidence Tables

Table 1: GRADE table for any web-based-based intervention

Patient or population: Caregivers
Intervention: Web-based interventions
Comparison: Control

Outcomes	**Anticipated absolute effects**[*] (95% CI)		№ of participants (studies) Quality of the evidence (GRADE)	Comments
	**Risk with Control**	**Risk with web-based interventions**		
Change in Self-esteem	-	SMD **0.85 SD higher** (0.12 higher to 1.57 higher)<br>32 (1 RCT) [h]	⊕◯◯◯ VERY LOW [i]	Assessed using 10-item Rosenberg Self-Esteem Scale, Scores may range from 10 to 4. Higher scores indicated greater self-esteem.
Change in Caregiver Strain	-	SMD **0.32 SD lower** (0.54 lower to 0.09 lower)	299 (1 RCT) [j]<br>⊕⊕⊕◯ MODERATE [b]	Assessed using Caregiver Strain Instrument, 14 self-report questions on a 5-point Likert scale, with answers ranging from 5 (strongly agree) to 0 (strongly disagree).

Multimedia Appendix 5. Detailed GRADE Evidence Tables

Table 1: GRADE table for any web-based-based intervention

Patient or population: Caregivers
Intervention: Web-based interventions
Comparison: Control

Outcomes	**Anticipated absolute effects*** (95% CI)		№ of participants (studies) Quality of the evidence (GRADE)	Comments	
	**Risk with Control**	**Risk with web-based interventions**			
SMD **0.1 SD lower** (0.66 lower to 0.45 higher) 152 (2 RCTs) [l]⨁◯◯◯ VERY LOW [b,c,d]Change in Social support<br><br>Assesses using 24-item Revised Memory and Behavior Problems Checklist (RMBPC, range; 0-96), CGs rate on a 5-point scale (0 = not at all; 4 = extremely) how much it 'bothered/upset' them.	-	SMD **0.38 SD lower** (1.12 lower to 0.35 higher)	64 (2 RCTs) [k]	⨁◯◯◯ VERY LOW [b,d]	Assessed using 6-item Lubben Social Network Scale (range: 5 to 11) in one study and 11-item Medical Outcomes Study Social Support Survey (range: 9 to 30) in the other study. Higher scores are better.
***The risk in the intervention group** (and its 95% confidence interval) is based on the assumed risk in the comparison group and the **relative effect** of the intervention (and its 95% CI).<br><br>**CI:** Confidence interval; **SMD:** Standardised mean difference -<br><br>Change in Reaction to problem behavior					

GRADE Working Group grades of evidence
High quality: We are very confident that the true effect lies close to that of the estimate of the effect
Moderate quality: We are moderately confident in the effect estimate: The true effect is likely to be close to the estimate of the effect, but there is a possibility that it is substantially different
Low quality: Our confidence in the effect estimate is limited: The true effect may be substantially different from the estimate of the effect
Very low quality: We have very little confidence in the effect estimate: The true effect is likely to be substantially different from the estimate of effect

Multimedia Appendix 5. Detailed GRADE Evidence Tables

Table 1: GRADE table for any web-based-based intervention

Patient or population: Caregivers
Intervention: Web-based interventions
Comparison: Control

Outcomes	**Anticipated absolute effects*** (95% CI)		№ of participants (studies)<br>Quality of the evidence (GRADE)	Comments	
	**Risk with Control**	**Risk with web-based interventions**			

**Quality assessment**							**№ of patients**		**Effect**	**Quality**	**Importance**
**№ of studies**	**Study design**	**Risk of bias**	**Inconsistency**	**Indirectness**	**Imprecision**	**Other considerations**	**Web-based Interventions**	**Control**	**Absolute (95% CI)**		
Change in Caregiver Burden											
5 [a]	randomised trials	serious [b]<br>not serious	serious [c]	very serious [d]	none	132	147	SMD **0.03 SD higher** (0.31 lower to 0.36 higher)	⊕◯◯◯<br>VERY LOW	CRITICAL	
Change in Self-efficacy / Mastery											
9 [e]	randomised trials	serious [b]	not serious	serious [c]	not serious	none	306	309<br>SMD **0.36 SD higher** (0.11 higher to 0.62 higher)	⊕⊕◯◯<br>LOW	CRITICAL	
Change in Life satisfaction											

Multimedia Appendix 5. Detailed GRADE Evidence Tables

Quality assessment							№ of patients		Effect	Quality	Importance
№ of studies	Study design	Risk of bias	Inconsistency	Indirectness	Imprecision	Other considerations	Web-based Interventions	Control	Absolute (95% CI)		
3 [f]	randomised trials	serious [b]	not serious	serious [c]	serious [g]	none	170	165	SMD **0.17 SD lower** (0.39 lower to 0.04 higher)	⨁◯◯◯ VERY LOW	CRITICAL
not serious not serious very serious [i] none 15 17 SMD **0.85 SD higher** (0.12 higher to 1.57 higher) ⨁◯◯◯ VERY LOW CRITICAL 1 [h] Change in Self-esteem											
Change in Caregiver Strainrandomised trials serious [i]											
not serious none 150 149 SMD **0.32 SD lower** (0.54 lower to 0.09 lower) ⨁⨁⨁◯ MODERATE CRITICAL not serious 1 [j] not serious											
Change in Social support randomised trials serious [b]											

Multimedia Appendix 5. Detailed GRADE Evidence Tables

Quality assessment							№ of patients		Effect	Quality	Importance
№ of studies	Study design	Risk of bias	Inconsistency	Indirectness	Imprecision	Other considerations	Web-based Interventions	Control	Absolute (95% CI)		
⨁◯◯◯ VERY LOW CRITICAL not serious not serious very serious [d] none 30 34 2 [k] SMD **0.38 SD lower** (1.12 lower to 0.35 higher)											
Change in Reaction to problem behavio rrandomised trials serious [b]											
not serious serious [c] very serious [d] none 71 81 SMD **0.1 SD lower** (0.66 lower to 0.45 higher) ⨁◯◯◯ VERY LOW CRITICAL 2 [l]											

randomised trials serious [b]

CI: Confidence interval; **SMD:** Standardised mean difference

Explanations

a. 1) Cristancho-Lacroix, 2015; 2) Hattink, 2015: 3) DuBenske, 2014; 4) Pagan-Ortiz, 2014; 5) Torkamani, 2014.
b. Serious concerns regarding risk of bias.
c. Serious concerns regarding clinical/methodological heterogeneity across studies due to differences in type and focus of e-technology interventions, length of intervention and informal caregiver population.
d. The sample size is <300 and effect estimate is imprecise.

e. 1) Beauchamp, 2005; 2) Núñez-Naveira, 2016: 3) Cristancho-Lacroix, 2015; 4) Hattink, 2015; 5) Nesbitt-Fowler, 2016; 6) Pagan-Ortiz, 2014; 7) Smith, 2012; 8) Hattink, 2016; 9) Kim, 2013.
f. 1) McLaughlin, 2013; 2) Núñez-Naveira, 2016; 3) DuBenske, 2014; 4) Pierce, 2009.
g. The effect estimate is imprecise.
h. Smith, 2012
i. Serious concerns for risk of bias and sample size <300.
j. Beauchamp, 2005
k. 1) Smith, 2012; 2) Pagan-Ortiz, 2014.
l. 1) Kajiyama, 2013; 2) Cristancho-Lacroix, 2015.

Multimedia Appendix 5. Detailed GRADE Evidence Tables

Table 2. GRADE table for web-based information or education interventions

Patient or population: Caregivers
Intervention: Web-based information or education
Comparison: Control

Outcomes	**Anticipated absolute effects**[*] (95% CI)		№ of participants (studies)	Quality of the evidence (GRADE)	Comments
	**Risk with Control**	**Risk with web-based Information or Education**			
Change in Self-efficacy / Mastery	-	SMD **0.31 SD higher** (0.08 higher to 0.53 higher)	299 (1 RCTs) [a]	⊕⊕⊕◯ MODERATE [b]	Assessed using 6-item self-efficacy (range: 6 to 42) on a 7-point type Likert scale. Higher scores indicating more feelings of competence.

Multimedia Appendix 5. Detailed GRADE Evidence Tables

Table 2. GRADE table for web-based information or education interventions

Patient or population: Caregivers
Intervention: Web-based information or education
Comparison: Control

Outcomes	Anticipated absolute effects* (95% CI)		№ of participants (studies)	Quality of the evidence (GRADE)	Comments
	Risk with Control	Risk with web-based Information or Education			
Change in Life Satisfaction	-	SMD **0.22 SD lower** (0.5 lower to 0.06 higher)	201 (1 RCT) [c]	⨁◯◯◯ VERY LOW [b,d]	Assessed using 5-item Satisfaction with Life (range 1-35) Scale. Higher scores indicate better outcome.

Multimedia Appendix 5. Detailed GRADE Evidence Tables

Table 2. GRADE table for web-based information or education interventions

Patient or population: Caregivers
Intervention: Web-based information or education
Comparison: Control

Outcomes	Anticipated absolute effects* (95% CI)		№ of participants (studies) Quality of the evidence (GRADE)	Comments
	Risk with Control	Risk with web-based Information or Education		
Change in Reaction to problem behavior	-	SMD **0.35 SD lower** (0.75 lower to 0.04 higher)	103 (1 RCT) [e]	⊕◯◯◯ VERY LOW [b,d] Assesses using 24-item Revised Memory and Behavior Problems Checklist (RMBPC, range; 0-96), CGs rate on a 5-point scale (0 = not at all; 4 = extremely) how much it 'bothered/upset' them.

Multimedia Appendix 5. Detailed GRADE Evidence Tables

Table 2. GRADE table for web-based information or education interventions

Patient or population: Caregivers
Intervention: Web-based information or education
Comparison: Control

Outcomes	**Anticipated absolute effects*** (95% CI)		№ of participants (studies) Quality of the evidence (GRADE)	Comments	
	**Risk with Control**	**Risk with web-based Information or Education**			
Change in Caregiver Strain	-	SMD **0.32 SD lower** (0.54 lower to 0.09 lower)	299 (1 RCT) [f]	⊕⊕⊕◯ MODERATE [b]<br><br>Assessed using Caregiver Strain Instrument, 14 self-report questions on a 5-point Likert scale, with answers ranging from 5 (strongly agree) to 0 (strongly disagree).	

*__The risk in the intervention group__ (and its 95% confidence interval) is based on the assumed risk in the comparison group and the **relative effect** of the intervention (and its 95% CI).

CI: Confidence interval; **SMD:** Standardised mean difference

GRADE Working Group grades of evidence
High quality: We are very confident that the true effect lies close to that of the estimate of the effect
Moderate quality: We are moderately confident in the effect estimate: The true effect is likely to be close to the estimate of the effect, but there is a possibility that it is substantially different
Low quality: Our confidence in the effect estimate is limited: The true effect may be substantially different from the estimate of the effect
Very low quality: We have very little confidence in the effect estimate: The true effect is likely to be substantially different from the estimate of effect

Multimedia Appendix 5. Detailed GRADE Evidence Tables

Quality assessment							№ of patients		Effect	Quality	Importance
№ of studies	Study design	Risk of bias	Inconsistency	Indirectness	Imprecision	Other considerations	Web-based Information or Education	Control	Absolute (95% CI)		
not serious not serious not seriousnone 150 149 SMD **0.31 SD higher** (0.08 higher to 0.53 higher) ⨁⨁⨁◯ MODERATECRITICAL 1 [a] Change in Self-efficacy / Mastery											
Change in Life Satisfa ctionra ndomis ed trials serious [b]											
very serious [d]none 104 97 SMD **0.22 SD lower** (0.5 lower to 0.06 higher) ⨁◯◯◯ VERY LOW CRITICAL not serious 1 [c] not serious											

Multimedia Appendix 5. Detailed GRADE Evidence Tables

Quality assessment							№ of patients		Effect	Quality	Importance
**№ of studies**	**Study design**	**Risk of bias**	**Inconsistenc y**	**Indirectness**	**Imprecision**	**Other considerations**	**Web-based Information or Education**	**Control**	**Absolute (95% CI)**		
Change in Reaction to problem behaviorrandomised trials serious [b]											
⊕◯◯◯ VERY LOW CRITICAL not serious not serious very serious [d]none 46 57 1 [e] SMD **0.35 SD lower** (0.75 lower to 0.04 higher)											
Change in Caregiver Strainrandomised trials serious [b]											
not serious not serious not serious none 150 149 SMD **0.32 SD lower** (0.54 lower to 0.09 lower) ⊕⊕⊕◯ MODERATE CRITICAL 1 [f]											

Multimedia Appendix 5. Detailed GRADE Evidence Tables

Quality assessment							№ of patients		Effect	Quality	Importance
№ of studies	Study design	Risk of bias	Inconsistency	Indirectness	Imprecision	Other considerations	Web-based Information or Education	Control	Absolute (95% CI)		

randomised trials serious [b]

CI: Confidence interval; SMD: Standardised mean difference
Explanations
a. Beauchamp, 2005
b. Serious concerns regarding risk of bias.
c. McLaughlin, 2013.
d. The sample size is <300 and effect estimate is imprecise.
e. Kajiyama, 2013
f. Beauchamp, 2005

Multimedia Appendix 5. Detailed GRADE Evidence Tables

Table 3: GRADE table for web-based information or education plus peer psychosocial support

Patient or population: Caregivers
Intervention: Web-based information or education plus peer psychosocial support
Comparison: Control

Outcomes **Anticipated absolute effects*** (95% CI)	№ of participants (studies)		Quality of the evidence (GRADE)	Comments	
	**Risk with Control**	**Risk with web-based Information or Education plus Peer Psychosocial Support**			
Change in Caregiver Burden	- SMD **0.17 SD higher** (0.24 lower to 0.57 higher)	95 (2 RCTs) [a]	⊕◯◯◯ VERY LOW [b,c]	Assessed using 1-item scale in one study and 22-item Zarit Burden Interview (Zarit) in the other study with a total range of 0 to 88, higher scores indicate greater burden.	

Multimedia Appendix 5. Detailed GRADE Evidence Tables

Table 3: GRADE table for web-based information or education plus peer psychosocial support

Patient or population: Caregivers
Intervention: Web-based information or education plus peer psychosocial support
Comparison: Control

Outcomes **Anticipated absolute effects*** (95% CI)	№ of participants (studies)		Quality of the evidence (GRADE)	Comments	
	**Risk with Control**	**Risk with web-based Information or Education plus Peer Psychosocial Support**			
Change in Self-efficacy / Mastery	- <br>SMD **0.14 SD higher** (0.41 lower to 0.69 higher)	156 (3 RCTs) [d]	⨁◯◯◯ VERY LOW [b,c]<br><br>Assessed using 7-item Short Sense of Competence Questionnaire (range: 7 to 35), Revised Scale for Caregiving Self-Efficacy (RSCS, range: 0 to 100) and Caregiver Competence Scale (range: 0 to 16).		

Multimedia Appendix 5. Detailed GRADE Evidence Tables

Table 3: GRADE table for web-based information or education plus peer psychosocial support

Patient or population: Caregivers
Intervention: Web-based information or education plus peer psychosocial support
Comparison: Control

Outcomes **Anticipated absolute effects*** (95% CI)	№ of participants (studies)		Quality of the evidence (GRADE)	Comments	
	**Risk with Control**	**Risk with web-based Information or Education plus Peer Psychosocial Support**			
Change in Life Satisfaction	-	SMD **0.08 SD higher** (0.43 lower to 0.58 higher)	61 (1 RCT) [e]	⨁◯◯◯ VERY LOW [b,c] Assessed using Revised Caregiving Satisfaction Scale, 6 items, range 0-30, higher scores in the RCSS indicate more feelings of satisfaction.	

Multimedia Appendix 5. Detailed GRADE Evidence Tables

Table 3: GRADE table for web-based information or education plus peer psychosocial support

Patient or population: Caregivers
Intervention: Web-based information or education plus peer psychosocial support
Comparison: Control

Outcomes **Anticipated absolute effects*** (95% CI)	№ of participants (studies)		Quality of the evidence (GRADE)	Comments	
	**Risk with Control**	**Risk with web-based Information or Education plus Peer Psychosocial Support**			
Change in Reaction to problem behavior	-	SMD **0.22 SD higher** (0.34 lower to 0.78 higher)	49 (1 RCT) [f]	⨁◯◯◯ VERY LOW [b,c] Assessed using Revised Memory and Behavior Problems Checklist (RMBPC) with 24 problems on 2 scales. A global score ranging from 0 to 4 was calculated for both scales.	

***The risk in the intervention group** (and its 95% confidence interval) is based on the assumed risk in the comparison group and the **relative effect** of the intervention (and its 95% CI).

CI: Confidence interval; **SMD:** Standardised mean difference

Multimedia Appendix 5. Detailed GRADE Evidence Tables

Table 3: GRADE table for web-based information or education plus peer psychosocial support

Patient or population: Caregivers
Intervention: Web-based information or education plus peer psychosocial support
Comparison: Control

Outcomes **Anticipated absolute effects*** (95% CI)	№ of participants (studies)		Quality of the evidence (GRADE)	Comments	
	**Risk with Control**	**Risk with web-based Information or Education plus Peer Psychosocial Support**			

GRADE Working Group grades of evidence
High quality: We are very confident that the true effect lies close to that of the estimate of the effect
Moderate quality: We are moderately confident in the effect estimate: The true effect is likely to be close to the estimate of the effect, but there is a possibility that it is substantially different
Low quality: Our confidence in the effect estimate is limited: The true effect may be substantially different from the estimate of the effect
Very low quality: We have very little confidence in the effect estimate: The true effect is likely to be substantially different from the estimate of effect

Quality assessment							№ of patients		Effect	Quality	Importance
**№ of studies**	**Study design**	**Risk of bias**	**Inconsistency**	**Indirectness**	**Imprecision**	**Other considerations**	**Web-based Information or Education + Peer Psychosocial Support**	**Control**	**Absolute (95% CI)**		
not serious not serious very serious [c]none 46 49 SMD **0.17 SD higher** (0.24 lower to 0.57 higher) ⨁◯◯◯ VERY LOW CRITICAL 2 [a] Change in Caregiver Burden											

Multimedia Appendix 5. Detailed GRADE Evidence Tables

Quality assessment							№ of patients		Effect	Quality	Importance
№ of studies	Study design	Risk of bias	Inconsistency	Indirectness	Imprecision	Other considerations	Web-based Information or Education + Peer Psychosocial Support	Control	Absolute (95% CI)		
Change in Self-efficacy / Mastery randomised trials serious [b]											
very serious [c]none 76 80 SMD **0.14 SD higher** (0.41 lower to 0.69 higher) ⨁◯◯◯ VERY LOW CRITICAL not serious 3 [d] not serious											
Change in Life Satisfactionrandomised trials serious [b]											
⨁◯◯◯ VERY LOW CRITICAL not serious not serious very serious [c]none 30 31 1 [e] SMD **0.08 SD higher** (0.43 lower to 0.58 higher)											

Multimedia Appendix 5. Detailed GRADE Evidence Tables

Quality assessment							№ of patients		Effect	Quality	Importance
**№ of studies**	**Study design**	**Risk of bias**	**Inconsistency**	**Indirectness**	**Imprecision**	**Other considerations**	**Web-based Information or Education + Peer Psychosocial Support**	**Control**	**Absolute (95% CI)**		
Change in Reaction to problem behaviorrandomised trials serious [b]											
not serious not serious very serious [c]none 25 24 SMD **0.22 SD higher** (0.34 lower to 0.78 higher) ⨁◯◯◯ VERY LOW CRITICAL 1 [f]											

randomised trials serious [b]

CI: Confidence interval; **SMD:** Standardised mean difference

Explanations

a. 1) Cristancho-Lacroix, 2015; 2) Hattink, 2015.
b. Serious concerns regarding risk of bias.
c. The sample size is <300 and effect estimate is imprecise.
d. 1) Cristancho-Lacroix, 2015; 2) Hattink, 2015; 3) Núñez-Naveira, 2016.
e. Núñez-Naveira, 2016

Multimedia Appendix 5. Detailed GRADE Evidence Tables

f. Cristancho-Lacroix, 2015

Table 4. GRADE table for web-based information or education plus professional psychosocial support

Outcomes

Patient or population: Caregivers
Intervention: Web-based information or education plus professional psychosocial support
Comparison: Control

Anticipated absolute effects* (95% CI) № of participants (studies)	Risk with Control	Risk with Internet-based Information or Education plus Professional Psychosocial Support
	-	SMD **1.2 SD higher** (0.48 higher to 1.92 higher)

***The risk in the intervention group** (and its 95% confidence interval) is based on the assumed risk in the comparison group and the **relative effect** of the intervention (and its 95% CI).

CI: Confidence interval; **SMD:** Standardised mean difference

GRADE Working Group grades of evidence
High quality: We are very confident that the true effect lies close to that of the estimate of the effect
Moderate quality: We are moderately confident in the effect

Table 4. GRADE table for web-based information or education plus professional psychosocial support

Outcomes

Patient or population: Caregivers
Intervention: Web-based information or education plus professional psychosocial support
Comparison: Control

**Anticipated absolute effects*** (95% CI) **№ of participants (studies)**	**Risk with Control**
	**Risk with Internet-based Information or Education plus Professional Psychosocial Support**
	**-** SMD **1.2 SD higher** (0.48 higher to 1.92 higher)

Quality assessment							№ of patients		Effect	Quality	Importance
**№ of studies**	**Study design**	**Risk of bias**	**Inconsistency**	**Indirectness**	**Imprecision**	**Other considerations**	**Web-based Information or Education + Professional Psychosocial Support**	**Control**	**Absolute (95% CI)**		
Change in Self-efficacy / Mastery											
1 [a]	randomised trials	serious [b]	not serious	not serious	very serious [c]	none	18	18	SMD **1.2 SD higher** (0.48 higher to 1.92 higher)	⨁◯◯◯ VERY LOW	CRITICAL

Confidence interval; **SMD:** Standardised mean difference

Multimedia Appendix 5. Detailed GRADE Evidence Tables

Table 4. GRADE table for web-based information or education plus professional psychosocial support

Outcomes

Patient or population: Caregivers
Intervention: Web-based information or education plus professional psychosocial support
Comparison: Control

**Anticipated absolute effects*** **(95% CI) № of participants (studies)**	**Risk with Control**	
	**Risk with Internet-based Information or Education plus Professional Psychosocial Support**	
	**-** SMD **1.2 SD higher** (0.48 higher to 1.92 higher)	

Table 5. GRADE table for web-based information or education plus peer and professional psychosocial support

Patient or population: Caregivers
Intervention: Web-based information or education plus professional psychosocial support
Comparison: Control

Outcomes	Anticipated absolute effects[*] (95% CI)		Relative effect (95% CI)	№ of participants (studies)	Quality of the evidence (GRADE)	Comments
	Risk with Control	Risk with web-based Information or Education plus Peer & Professional Psychosocial Support				
Change in Caregiver Burden	-	SMD **0.03 SD lower** (0.57 lower to 0.5 higher)	-	184 (3 RCTs) [a]	⨁◯◯◯ VERY LOW [b,c]	Assessed using 22-item Zarit Burden Interview (Zarit) with a total range of 0 to 88 in two studies & Caregiver Quality of Life (CQOLC) Burden Subscale (10 Likert-type items) in one study.
Change in Self-efficacy / Mastery	-	SMD **0.52 SD higher** (0.1 higher to 0.94 higher)	-	92 (3 RCTs) [d]	⨁◯◯◯ VERY LOW [e]	Assessed using 9-item Pearlin Mastery Scale with range 9 to 30 in two studies and the General Self-Efficacy scale with 8-items on a 1 to 5 scale (range 8 to 40) in one study.
Change in Life satisfaction	-	SMD **0.24 SD lower** (0.7 lower to 0.22 higher)	-	73 (1 RCT) [f]	⨁◯◯◯ VERY LOW [b,c]	Assessed using Satisfaction with Life Scale, 5 items rated on a scale from 1 to 7 (range 1 to 35).
Change in Self-esteem	-	SMD **0.85 SD higher** (0.12 higher to 1.57 higher)	-	32 (1 RCT) [g]	⨁◯◯◯ VERY LOW [e]	Assessed using 10-item Rosenberg Self-Esteem Scale, Scores may range from 10 to 4. Higher scores indicated greater self-esteem.
Change in Social support	-	SMD **0.38 SD lower** (1.12 lower to 0.35 higher)	-	64 (2 RCTs) [h]	⨁◯◯◯ VERY LOW [b,c]	Assessed using 6-item Lubben Social Network Scale (range: 5 to 11) in one study and 11-item Medical Outcomes Study Social Support Survey (range: 9 to 30) in the other study. Higher scores are better.

***The risk in the intervention group** (and its 95% confidence interval) is based on the assumed risk in the comparison group and the **relative effect** of the intervention (and its 95% CI).

CI: Confidence interval; **SMD:** Standardised mean difference

[illegible]

Multimedia Appendix 5. Detailed GRADE Evidence Tables

Quality assessment							№ of patients		Effect	Quality	Importance
**№ of studies**	**Study design**	**Risk of bias**	**Inconsistency**	**Indirectness**	**Imprecision**	**Other considerations**	**Web-based Information or Education + Peer & Professional Psychosocial Support**	**Control**	**Absolute (95% CI)**		
Change in Caregiver Burden											
3 [a]	randomised trials	serious [b]	not serious	not serious	very serious [c]	none	86	98	SMD **0.03 SD lower** (0.57 lower to 0.5 higher)	⨁◯◯◯ VERY LOW	CRITICAL
Change in Self-efficacy / Mastery											
3 [d]	randomised trials	serious [b]	not serious	not serious	very serious [e]	none	45	47	SMD **0.52 SD higher** (0.1 higher to 0.94 higher)	⨁◯◯◯ VERY LOW	CRITICAL
Change in Life satisfaction											
1 [f]	randomised trials	serious [b]	not serious	not serious	very serious [c]	none	36	37	SMD **0.24 SD lower** (0.7 lower to 0.22 higher)	⨁◯◯◯ VERY LOW	CRITICAL
Change in Self-esteem											
1 [g]	randomised trials	serious [g]	not serious	not serious	very serious [e]	none	15	17	SMD **0.85 SD higher** (0.12 higher to 1.57 higher)	⨁◯◯◯ VERY LOW	CRITICAL
Change in Social support											
2 [h]	randomised trials	serious [b]	not serious	not serious	very serious [c]	none	30	34	SMD **0.38 SD lower** (1.12 lower to 0.35 higher)	⨁◯◯◯ VERY LOW	CRITICAL

Multimedia Appendix 5. Detailed GRADE Evidence Tables

CI: Confidence interval; **SMD:** Standardised mean difference

Explanations

a. 1) DuBenske, 2014; 2) Pagan-Ortiz, 2014; 3) Torkamani, 2014.
b. Serious concerns regarding risk of bias.
c. The sample size is <300 and effect estimate is imprecise.
d. 1) Smith, 2012; 2) Pagan-Ortiz, 2014; 3) Nesbitt-Fowler, 2016.
e. Serious concerns for risk of bias and sample size <300.
f. Pierce, 2009
g. Smith, 2012
h. 1) Smith, 2012; 2) Pagan-Ortiz, 2014.

Multimedia Appendix 5. Detailed GRADE Evidence Tables

Table 6. GRADE table for web-based information or education plus professional psychosocial support plus electronic monitoring

Patient or population: Caregivers
Intervention: Technology (web + telephone: Monitoring + Peer & Professional psychosocial support)
Comparison: Control

Outcomes	**Anticipated absolute effects*** (95% CI)		№ of participants (studies)	Quality of the evidence (GRADE)	Comments
	**Risk with Control**	**Risk with web-based Information or Education plus Professional Psychosocial Support plus Electronic Monitoring**			
Change in Self-efficacy / Mastery	-	SMD **0.17 SD higher** (0.52 lower to 0.87 higher)	32 (1 RCT) [a]	⨁◯◯◯ VERY LOW [b,c]	Assessed using Caregiver Competence Scale, with a maximum score of 16 points and higher scores indicating more feelings of competence.

***The risk in the intervention group** (and its 95% confidence interval) is based on the assumed risk in the comparison group and the **relative effect** of the intervention (and its 95% CI).

CI: Confidence interval; **SMD:** Standardised mean difference

GRADE Working Group grades of evidence
High quality: We are very confident that the true effect lies close to that of the estimate of the effect
Moderate quality: We are moderately confident in the effect estimate: The true effect is likely to be close to the estimate of the effect, but there is a possibility that it is substantially different
Low quality: Our confidence in the effect estimate is limited: The true effect may be substantially different from the estimate of the effect
Very low quality: We have very little confidence in the effect estimate: The true effect is likely to be substantially different from the estimate of effect

Multimedia Appendix 5. Detailed GRADE Evidence Tables

Quality assessment							№ of patients		Effect	Quality	Importance
**№ of studies**	**Study design**	**Risk of bias**	**Inconsistency**	**Indirectness**	**Imprecision**	**Other considerations**	**Web-based Information or Education + Professional Psychosocial Support + Electronic Monitoring**	**Control**	**Absolute (95% CI)**		
Change in Self-efficacy / Mastery											
1 [a]	randomised trials	serious [b]	not serious	not serious	very serious [c]	none	17	15	SMD **0.17 SD higher** (0.52 lower to 0.87 higher)	⨁◯◯◯ VERY LOW	CRITICAL

CI: Confidence interval; **SMD:** Standardised mean difference

Explanations

a. Hattink, 2016
b. Serious concerns regarding risk of bias.
c. The sample size is <300 and effect estimate is imprecise.
